# Anti‐seasonal flooding drives substantial alterations in riparian plant diversity and niche characteristics in a unique hydro‐fluctuation zone

**DOI:** 10.1002/ece3.70036

**Published:** 2024-08-09

**Authors:** Ye Liu, Xiaodie Duan, Xiaoling Li, Wenxiong Yi, Gong Chen, Jin Yang, Danli Deng, Xiaojuan Guo, Zhengjian Yang, Guiyun Huang, Meixiang Hu, Chen Ye

**Affiliations:** ^1^ Engineering Research Center of Eco‐Environment in Three Gorges Reservoir Region, Ministry of Education, Hubei International Scientific and Technological Center of Ecological Conservation and Management in the Three Gorges Area, College of Biological and Pharmaceutical Science China Three Gorges University Yichang Hubei China; ^2^ Rare Plants Research Institute of Yangtze River China Three Gorges Corporation Yichang China; ^3^ Key Laboratory of Aquatic Botany and Watershed Ecology Wuhan Botanical Garden, Chinese Academy of Sciences Wuhan China

**Keywords:** anti‐seasonal flooding, niche characteristics, plant guilds, riparian ecosystems, species diversity pattern, Three Gorges Reservoir

## Abstract

Human‐induced disturbances such as dam construction and regulation have led to widespread alterations in hydrological processes and thus substantially influence plant characteristics in the hydro‐fluctuation zones (HFZs). To reveal utilization of limited resources and mechanisms of inter‐specific competition and species co‐existence of plant communities based on niche breadth and overlap under the different HFZs of the Three Gorges Reservoir (TGR) in China, we conducted a field investigation with 368 quadrats on the effects of hydrological alterations on plant diversity and niche characteristics. The results showed anti‐seasonal flooding precipitated the gradual disappearance of the original diverse niches, resulting in the reduction of plant species richness and functional diversity and more obvious competition among plant species with similar resource requirements. Annuals, perennials and shrubs accounted for 71.23%, 27.39% and 1.37%, respectively, suggesting that annuals and flood‐tolerant riparian herbs were favored under such novel flooding conditions. A consistent increase in species number, Shannon‐Wiener diversity index and Simpson dominance index with altitude was inconsistent with hump‐shaped diversity–disturbance relationship of the intermediate disturbance hypothesis, while the opposite trend was observed for the Pielou evenness index. This species distribution pattern might be caused by several synergetic attributes (e.g., the submergence depth, plant tolerant capacity to flooding, life form, dispersal mode and inter‐specific competition). Vegetation types shifted from xerophytes to mesophytes and eventually to hygrophytes with the increasing flooding time in the HFZs. Hydrological alterations proved to be the paramount driver of vegetation distribution in the different HFZs. The niche analysis provided the first insights on the mechanisms of resource utilization and inter‐specific competition, of which annuals could germinate quickly after soil drainage to achieve the greatest competitive advantages and occupy a larger niche space than other plants. Vegetation was still in the early stage of primary succession in the novel riparian forests. Therefore, vegetation restoration strategies should be biased towards herbaceous plants, due to annuals with better environmental adaptability, supplemented by shrubs and small trees. To establish a complete reference system for vegetation restoration, natural vegetation monitory plots in the different succession stages should be established in the different HFZs of the TGR, and their environmental conditions, community structures and inter‐specific relationships further analyzed.

## Graphical abstract

The anti‐seasonal water level fluctuation significantly reduced the species diversity of the hydro‐fluctuation belt.

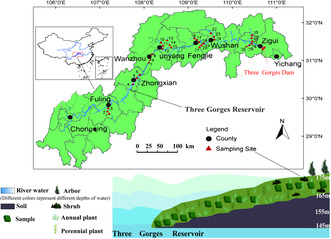

## 1 INTRODUCTION

Global warming and anthropogenic disturbance such as dam constructions are associated with an increase in flooding events, making many ecosystems worldwide vulnerable to submergence and flooding (Laloë et al., 2021; Pucciariello & Perata, 2012; Xiao et al., 2022), which are major abiotic stresses for plants as significant determinants of plant species distribution worldwide, functions of plant guilds, forest community composition, structure and dynamics (Arif et al., 2024; Jackson & Colmer, 2005). Hirabayashi et al. (2013) also reported that a warmer climate would increase the risk of floods. What is more, alterations in hydrological regimes and floods owing to the joint effects of operation of reservoirs and warmer climates play an important role in regulating the foristic composition, species diversity patterns and niche characteristics of riparian forest (Jian et al., 2017; Su et al., 2020). As the floodwaters move onto the riparian area, the soils became hypoxic, even resulting in severe anaerobism for plant roots, which posing a threat to the distribution of riparian forest plants without specific traits to adapt to anaerobic condition and ultimately lead to plant death (Feng et al., 2020; Gibbs & Greenway, 2003). Consequently, the unnaturally flooding (winter flooding) and prolonged inundation duration (nearly half a year) would remarkably influence the plant guilds, the plant community composition, species diversity pattern, niche characteristics and patterns of resource utilization of riparian forest of the Three Gorges Reservoir (TGR) in China. Therefore, it has crucial ecological significance to evaluate influences of anti‐seasonal long flooding on the plant guilds and niche characteristics of plant communities in the newly formed terrestrial–aquatic interfaces for vegetation restoration and reconstruction in the riparian forest. However, the responses of riparian vegetation by the niche characteristics to such unnaturally continuous flooding environment were less documented.

To comprehend the resource utilization patterns and ecological adaptability of diverse plant populations across varied environments and to furnish insights for biodiversity conservation and the restoration of degraded riparian forest ecosystems, the niche theory has been invoked and stands as a pivotal framework explaining species coexistence and competition within natural plant communities (Cui et al., 2013; D'Andrea et al., 2020; Jian et al., 2017; Slatyer et al., 2013). Niche involves two complementary aspects. One relates to the space occupied by a group of species or a guild in the ecological space and the other relates to resource utilization and competition among coexisting species (Lengyel et al., 2020). Niche breadth measures the range of resource characteristics across which a species exists and indicates the extent that species utilize different types of resources. Niche overlap has often been used as a measure of potential competition between species (Pastore et al., 2021), because it is expected to determine how many and which species can coexist in a guild. Previous studies found that species with similar patterns of resource utilization (i.e., species of the same guilds) were susceptible to competitive interactions that affect the community structure (Pérez‐Crespo et al., 2013). Patterns of resource utilization (either food or habitat resources) were normally analyzed in the framework of niche theory, i.e., members of the same guilds similarly exploit similar resources and may be underlying competitors. It is generally agreed that the number of related species that can coexist in a given community depends on the niche widths of the several species and the degree to which their niches overlap (Sánchez González et al., 2023). To explore ecological processes, such as competition over shared resources, both niche breadth and niche overlap provide indirect ways (Eurich et al., 2018). Moreover, the environmental impacts on the plant community also reflect the adaptation and evolution of the plant community to its conditions. Plant communities that inhabit aquatic ecosystems usually have a complex structure driven by a large number of variables that influence plant–plant interactions (Arnst, 2013). So, it is necessary to evaluate the available resources and the relative amounts of inter‐ and intra‐specific competition by niche width and overlap among the coexisting species of the community in specific environments such as in a novel hydro‐fluctuation zone.

As the largest hydropower project in the world, the Three Gorges Dam (TGD) on the Yangtze River was initiated in 1994 (Zhang et al., 2013), and first impoundment occurred in June 2003 (Yang, Chen, et al., 2012; Yang, Liu, et al., 2012). The water level of the reservoir fluctuates from 145 m in summer (May to September) to 175 m in winter (October to April), resulting in the formation of the hydro‐fluctuation zones (HFZs) with an area of 350 km^2^ in the reservoir (Jiajia et al., 2023; Ye et al., 2013). The hydrological regime of the Three Gorges Reservoir (TGR) was the exact opposite of the natural flood rhythms of the Yangtze River, which formed a unique riparian ecosystem. Before the impoundment of the TGR, the main vegetation types of riparian forests in the zone below an elevation of 175 m were trees (e.g., *Pinus massoniana* and *Cupressus funebris*), shrubs (e.g., *Vitex negundo*, *Securinega suffruticosa* and *Myricaria laxiflora*) and herbs (*Imperata cylindrica*, *Arthraxon hispidus* and *Cynodon dactylon*) (Chen et al., 2008). After the filling of the TGR, the reversal of submergence time and prolonged inundation duration precipitated the loss of previous vegetation, and annual plants such as *Setaria viridis*, *Digitaria ciliaris*, and *Comnyza canadensis* currently became dominant species (Lu et al., 2010; Ye et al., 2013). According to the plant species distribution surveys from the HFZs in the TGR in 2009, the decreased ratio of plant families, genera, and species of post‐dam riparian vegetation in 2009 was 26.51%, 29.58% and 42.96%, respectively, compared with the pre‐dam riparian plant species in 2001 (Liu et al., 2011). The vegetation investigation of the Pengxi River, Baijia Stream and Xiangxi River in the TGR also showed that the vegetation in the HFZs of the TGR was seriously degenerated, the community structure was single and the species richness and diversity decreased (Li et al., 2022; Liu et al., 2011; Xiang et al., 2020; You et al., 2017; Yuan et al., 2014). The hydrological alterations greatly degraded biodiversity in the TGR disturbance zone, particularly the disappearance of indigenous vegetation significantly would undermine the function of the regional ecosystem service (Zhu et al., 2020). Vegetation restoration in the HFZs has become an increasing concern in recent years (Gong et al., 2023; Ye et al., 2013; Zhang et al., 2016). Thus, it is important to understand the plant community characteristics in the novel riparian forest. However, there was a lack of application of niche theory to explore its formation mechanism of plant community, which is always a key issue for plant community ecologist.

Current research on the biological community structure and diversity of drawdown zones has largely focused on plant community diversity and its distribution patterns, leading to a fairly in‐depth understanding of these aspects (Su et al., 2020; Ye et al., 2013). However, little is known on how anti‐seasonal and continuous flooding hydrological alterations affect the riparian guilds, from functional and niche perspectives, of the unique drawdown zone vegetation after 19 times of operation of the TGR.

The TGR catchment provides a unique scenario to investigate the functional responses of riparian herbaceous species to the novel anti‐seasonal and continuous flooding environments across a 600 km stream gradient in a globally‐significant river system. We randomly selected 30 reaches along the shorelines of the TGR subjected to anti‐seasonal and continuous flooding. Fieldwork was conducted after 19 times of operation of the TGR to answer the following questions. (1) What were specific plant community characteristics and functional diversity in the unique riparian ecosystem after 19 times of operation of the TGR? (2) Which plant guilds were favored or disfavored by the novel anti‐seasonal and continuous flooding environment? and (3) How did the niche characteristics of dominant species change in various flooding environments, especially along an elevation gradient in the TGR? Accordingly, we hypothesized that (1) anti‐seasonal flooding substantially altered the plant community characteristics and species diversity patterns, and reduced species diversity in the novel riparian ecosystem of the TGR, (2) the more niche breadth of dominant plant species in the unique riparian ecosystem would imply that they would utilize more limited resources and the more niche overlap would show the more inter‐specific competition and co‐existence in the plant community and (3) anti‐seasonal and continuous flooding would precipitate the gradual disappearance of the original diverse niches, resulting in more uniform habitats, and there was obvious competition among species with similar resource requirements. This work will help to provide optimal plant guild selection and scientific plant configuration for ecological restoration efforts in the novel riparian forest of the TGR region and the similar ecologically fragile areas.

## 2 MATERIALS AND METHODS

### 2.1 Study area

**FIGURE 1:**
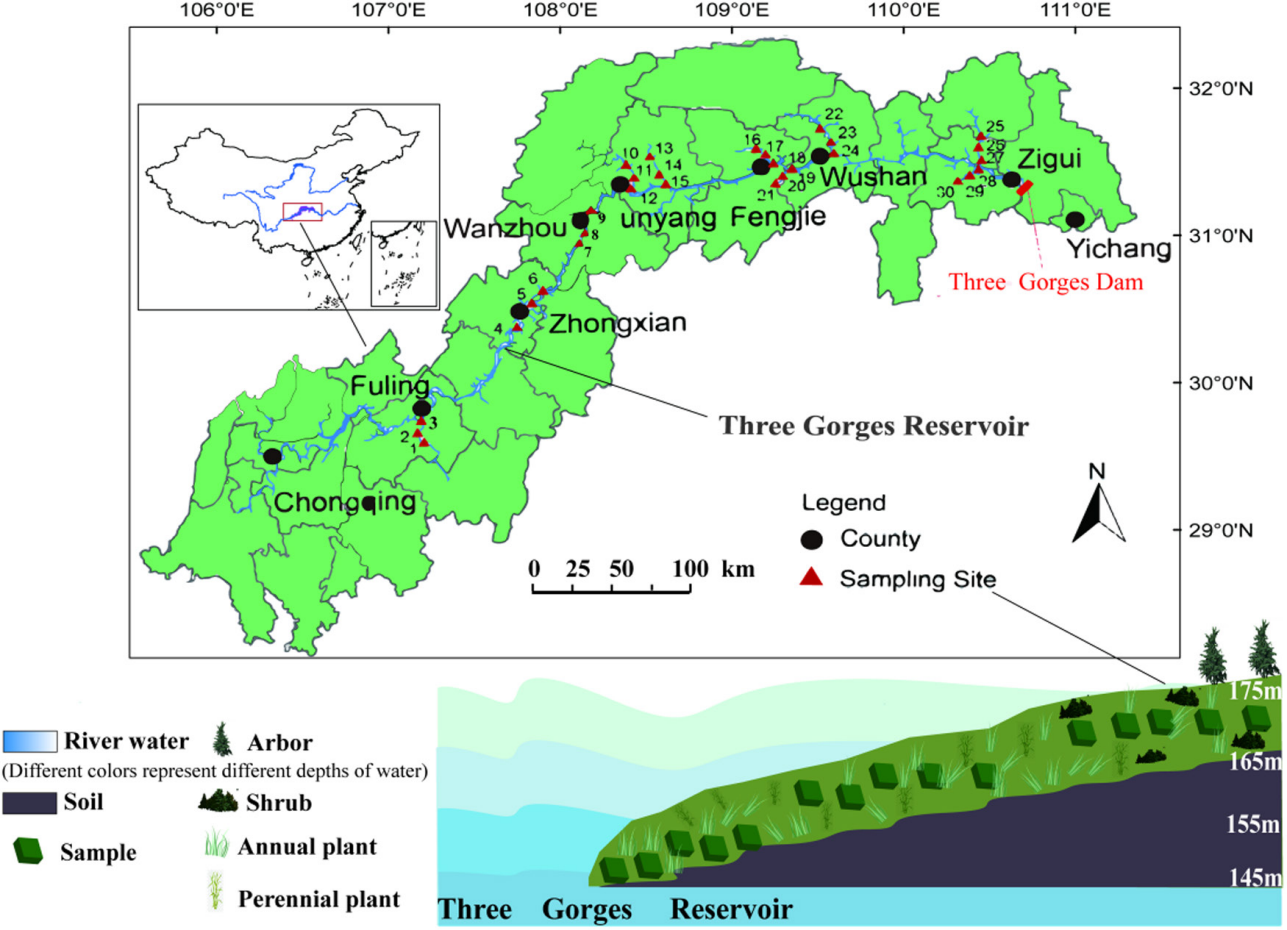
The study area in the water‐level‐fluctuation zone of the TGR, China. 1–3. Wujiang; 4–6. Zhongxian; 7–9. Wanzhou; 10–12. Pengxi River; 13–15. Tangxi River; 16–18. Meixi River; 19–21. Daxi River; 22–24. Daning River; 25–27. Tongzhuang River; 28–30. Xiangxi River.

The TGR region (105°49′~110°50′ E, 28°31′~31°25′ N) lies in a 660‐km valley from Yichang to upstream Chongqing, China (Figure 1). This area is characterized by a subtropical monsoon climate, with an annual mean temperature of 16.5–19.0°C and annual mean precipitation ranges from 886 to 1614 mm, 80% of which occurs between April and October (Ye et al., 2011). The soil is purple soil consisted of 29% sand, 49% silt and 22% clay in the top 20 cm. Our study area is located in the water level fluctuation zone of the TGR, where the water level fluctuates from 145 m a.s.l. in summer to 175 m a.s.l. in winter (Ye et al., 2015). Summer is the rain season in the study area which results in seasonal water level fluctuations that temporally range between 145 and 155 m with flooded periods lasting from a few days to approximately two weeks (Wang et al., 2014). However, due to the regulation of the TGR, the highest water level rise occurs in winter and falls to the lowest level in summer in an annual cycle. These changes in water level fluctuation are opposite to natural seasonal fluctuations and are called “anti‐seasonal” (Willison et al., 2013). The duration of anti‐seasonal flood differs according to elevation (i.e., 145–155 m a.s.l. with an average inundation duration of 286 days per year; 155–165 m a.s.l., with an average inundation duration of 237 days per year; and 165–175 m a.s.l., with an average inundation of 169 days per year). Refer hydrological alterations at this website (https://cj.msa.gov.cn/xxgk/xxgkml/aqxx/swgg/).

### 2.2 Vegetation investigation

Investigation was conducted in August to September, just before the water level rise in the TGR. At this time of the year, the vegetation in the HFZs was exposed and maximally recovered from the preceding flood, which occurred in the summer, autumn, and winter in the HFZs. Sample transects with 50 m lengths were defined and were parallel to the water level gradient. Within the transects, 30 reaches were randomly selected along the shorelines of the TGR subjected to anti‐seasonal and continuous flooding from upstream to downstream in the Reservoir in June–September, 2022. Among them, 24 sampling sites were located in the tributaries of the Yangtze River (1–3; 10–30) and 6 sampling sites were located in the mainstream (4–9). At each sampling site we established a transect along an elevation gradient, from 145 to 175 m a.s.l. (i.e., bottom, 145–155 m; middle, 155–165 m; and top, 165–175 m). Because flooding regime varies based on elevation, these transects allowed us to investigate the effects of flooding on riparian ecosystem properties. Five 1 × 1 m herb quadrats were investigated in the three sections of each sampling site, the relative density, relative frequency, relative height and relative coverage. However, survey was only carried out between the elevations of 156–165; 166–175 m in Wujiang River and Zhongxian because of high water level during the sampling period. Therefore, a total of 368 plant quadrats were investigated. Each vegetation sample quadrat was a focal point for flora survey and the categorical traits were obtained. Plant species were identified according to the Flora Reipublicae Popularis Sinicae (http://www.iplant.cn/frps). All plant species were inventoried in each quadrat. For each species in a quadrat, we recorded all the individuals of a given species in each quadrat, measured the average height of 10 randomly selected individuals and recorded the coverage by estimated the projective canopy area.

The soil samples (0–20 cm) were investigated using the cut ring method. Three random topsoil samples (0–20 cm) were collected and then mixed to form a composite sample at each elevation zone of each transect. A total of 129 mixed soil samples were sealed in plastic bags and brought to the laboratory. Soil samples were air‐dried and sieved (<2 mm) before analysis. The sketch map of study area and sampling sections is shown in Figure 1.

### 2.3 Environmental variable analysis

Soil pH was determined with a soil to water ratio of 1:5, using a combination glass electrode (Kabała & Musztyfaga, 2015). Soil organic matter (OM) content was determined by potassium dichromate titrimetric solution with the method detection limit (MDL) of 0.5 g kg^−1^. TN (total nitrogen) was determined by the semi‐micro Kjeldahl method. TP (total phosphorus) and AP (available phosphorus) were measured by molybdenum‐antimony anti‐spectrophotometric method. TK (total potassium) and AK (available potassium) were determined by flame photometric method (Bao, 2000). A 15‐g sample of soil was extracted by shaking with 100 mL of 2 M KCl for 1 h. Exchangeable ${\mathrm{NH}}_{4}^{+}‐N$ and ${\mathrm{NO}}_{3}^{-}‐N$ were determined with spectrophotometer using the Indophenol blue colorimetric method and Phenol disulfonic acid colorimetry, respectively (Ye et al., 2015). The heterogeneity of soil environmental factors in different elevations in the TGR is shown in Figure 7.

### 2.4 Data analysis

#### 2.4.1 Importance value

The importance values of different species at a given community were calculated on the basis of the relative coverage (RC), relative frequency (RF), relative height (RH) and the relative density (RD) of each species in different quadrats and then used as the indicators to determine the dominant herbaceous plant species in the HFZs and the niche measurement. The calculation formula is

(1) $$P_{i}=\left(\mathrm{RC}+\mathrm{RH}+\mathrm{RF}+\mathrm{RD}\right)/4$$

where *P*_*i*_ is the importance value of the *i*‐th species, RC is the projective coverage of a given species divided by the total coverage of all the species in all the quadrats; RF is number of occurrence of the species divided by the total number of occurrence of all the species in all the quadrats, RH is average height of the species divided by the total height of all the species in all the quadrats and RD is the total number of individuals of the species divided by the total number of all the species in all the quadrats.

#### 2.4.2 Species diversity index

Fisher's α‐diversity index was characterized by species number (*S*), Shannon diversity index (*H*), Pielou evenness index (*E*), and Simpson dominance index (*D*). The calculation formulas are (Curtis & McIntosh, 1951).

(2) $$\text{Shannon diversity index}:H=-\Sigma P_{i}\ln P_{i}$$

(3) $$\text{Pielou evenness index}:E=\frac{H}{\ln S}$$

(4) $$\text{Simpson}\;\text{dominance}\;\text{index}:\;H=-\sum P_{i}^{2}$$

where *P*_*i*_ is the importance value of the *i*‐th species and *S* is the number of species appearing within the quadrat.

#### 2.4.3 Niche breadth

The niche breadth, as proposed by Levin, was calculated by Colwell's modified formula (Feinsinger et al., 1981):

(5) $$B_{i}=\frac{1}{r\times \sum_{h=1}^{r}{\left(P_{\mathrm{ih}}\right)}^{2}}$$

where *B*_*i*_ is the niche breadth of the *i*‐th species, *P*_*ih*_ is the ratio of the importance value of the *i*‐th species at the *h*‐th resource level to the sum of the importance values of the species at all resource levels, *r* is the number of resource levels, and the value range is [0, 1].

#### 2.4.4 Niche overlap

The Pianka formula was used to calculate niche overlap (Pianka, 1974).

(6) $$O_{\mathrm{ih}}=\frac{\int_{h=1}^{r}P_{\mathrm{ih}}P_{\mathrm{jh}}}{\sqrt{\int_{h=1}^{r}P_{\mathrm{ih}}^{2}\int_{h=1}^{r}P_{\mathrm{jh}}^{2}}}$$

where *O*_*ij*_ is the niche overlap of populations *i* and *j*, and *p*_*ih*_ and *p*_*ij*_ are the proportion of the importance values of the *i*‐th and the *j*‐th species at the *h*‐th resource level in the synthesis of the importance values of the species at all resource levels, and *r* is the number of resource levels, and the range of the formula is [0, 1].

#### 2.4.5 The total mean of niche overlap values

The total mean value of niche overlap among all communities (TAO_*ih*_) in the sample land is calculated as follows (Chen et al., 2019):

(7) $${\mathrm{TAO}}_{\mathrm{ih}}=\frac{{\text{TO}}_{\mathrm{ih}}}{\mathrm{TP}}$$

where TO_*ih*_ is the total number of niche overlap values among all populations in the quadrat and TP is the total species logarithm.

#### 2.4.6 Statistical analysis

Two‐way clustering analysis provides a very visual picture of the distribution, classification, and degree of similarity between species in a community. In this study, we conducted clustering analysis of plant species in the surveyed quadrat, and calculated the significant values of the species in the sample to obtain the plant significant value matrix as the basis of clustering analysis according to the program method in “Quantitative Ecology‐Application of the R Language”. The groups were designated as riparian guilds where each vegetation group comprising a guild: (1) contains species sharing similar features and (2) shares a similar environment.

Canonical correspondence analysis (CCA) was used to analyze the data of 10 environmental factors from 10 sample sites in the TGR area and 31 plant species after excluding species with frequency less than 3 from the sample sites to get information such as the structure of the biological community, the relationship between the biological community and environmental factors. We conducted CCA using the CANOCO package v.5.0 (Ithaca, NY, USA). In the present investigation, univariate analysis of variance was conducted using SPSS version 22.0. Subsequently, the graphical representation of data, including the construction of histograms, box plots, and linear regression analyses, was executed utilizing Origin 2018 software on a Windows platform. Two‐way clustering analysis used the program packages such as vegan, gclus and cluster in R software based on investigation in 2022.

## 3 RESULTS

### 3.1 Foristic composition

A total of 73 vascular plant species were identified, belonging to 65 genera of 25 families, including 52 species of annual herbs, 20 species of perennial herbs and 1 species of shrub, accordingly accounting for 71.23%, 27.39% and 1.37% of the total number of species, respectively. The plant families with the largest number of species in the studied area were Compositae (*N* = 15, 20.56%), Gramineae (*N* = 12, 16.45%), Leguminosae (*N* = 9, 12.32%), and Euphorbiaceae (*N* = 5, 8.22%), respectively. A significant proportion of plants of single genus and species in the survey area were in 13 families (i.e., 13 genera and 13 species), representing 52% of the total number of families, 20% of the total number of genus and 17.8% of the total number of species, respectively.

**FIGURE 2:**
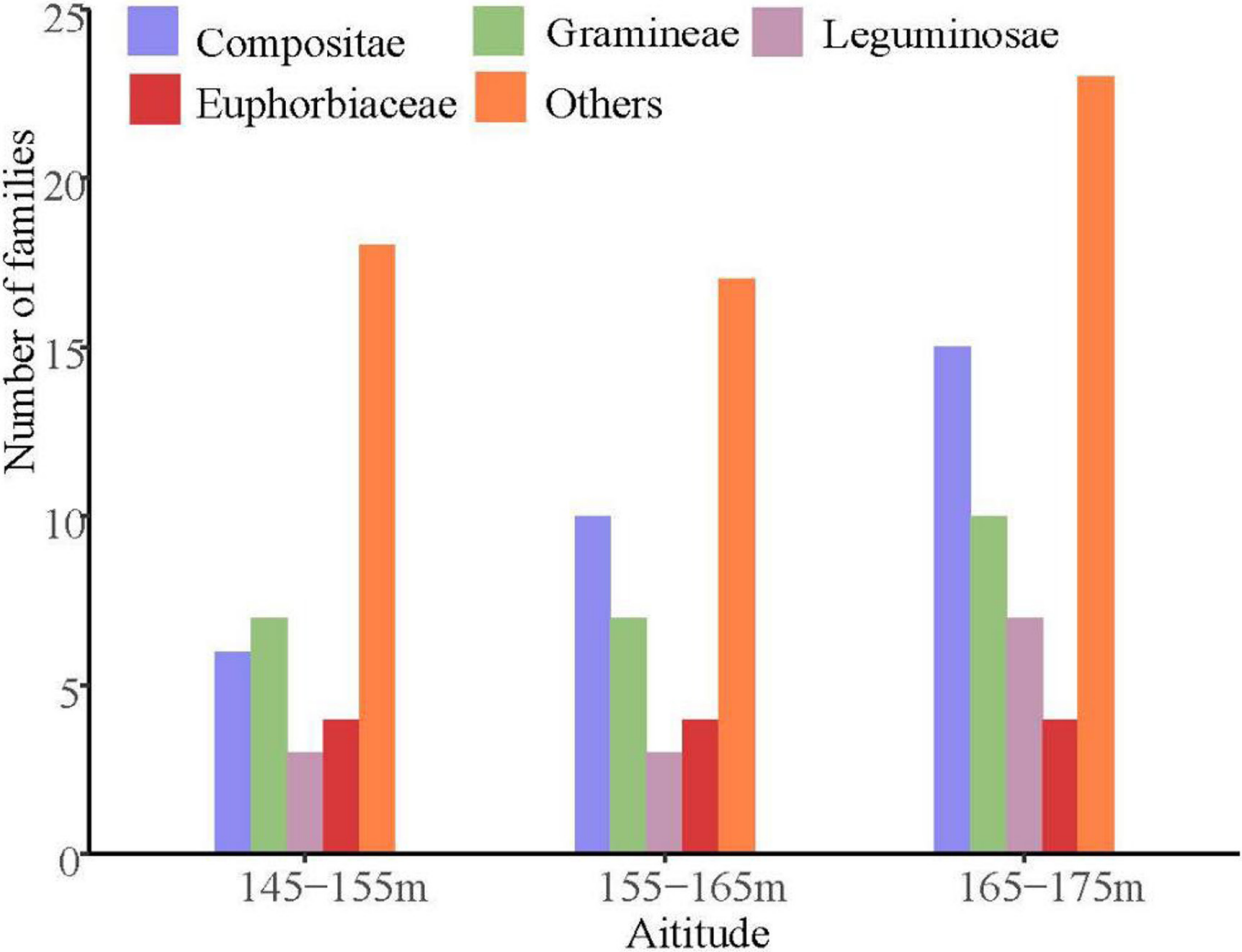
Families composition among three altitude sections in the water level fluctuation zone of the TGR. (*n* = 105 for 145–155 m; *n* = 132 for 155–165 m; *n* = 129 for 165–175 m).

There were different plant species, genera, and families at different altitudes (145–155, 155–165, and 165–175 m) in the WLFZ of the TGR. Individually, there were 38 vascular plant species belonging to 32 genera of 16 families at the 145–155 m, 43 vascular plant species belonging to 39 genera of 19 families at the 155–165 m and 57 vascular plant species belonging to 50 genera of 18 families at the 165–175 m (Figure 2). Species numbers significantly increased with increasing elevation. Annual herbs was the dominant life form in the whole study area, accounting for 78.95% of plant species at the elevation intervals of 144–155 m, 74.42% at the altitudes of 155–165 m and 71.93% at the altitudes of 165–175 m, respectively. The less frequent life form was perennial herb (145–155 m. *n* = 7, 18.42%; 155–165 m. *n* = 11, 25.58%; 165–175 m. *n* = 16, 28.07%). Compositae and Gramineae were the larger families with 6–15 species than other ones and contributed to 37.01% of the total plant species in the current study (Figure 2). The proportion of Compositae and Gramineae plants showed an upward trend with the increasing altitude. The proportion of annual herbs showed a decreased trend along altitude gradients, whereas the proportion of perennial herb species showed an opposite trend.

### 3.2 Spatial variation in plant diversities

**FIGURE 3:**
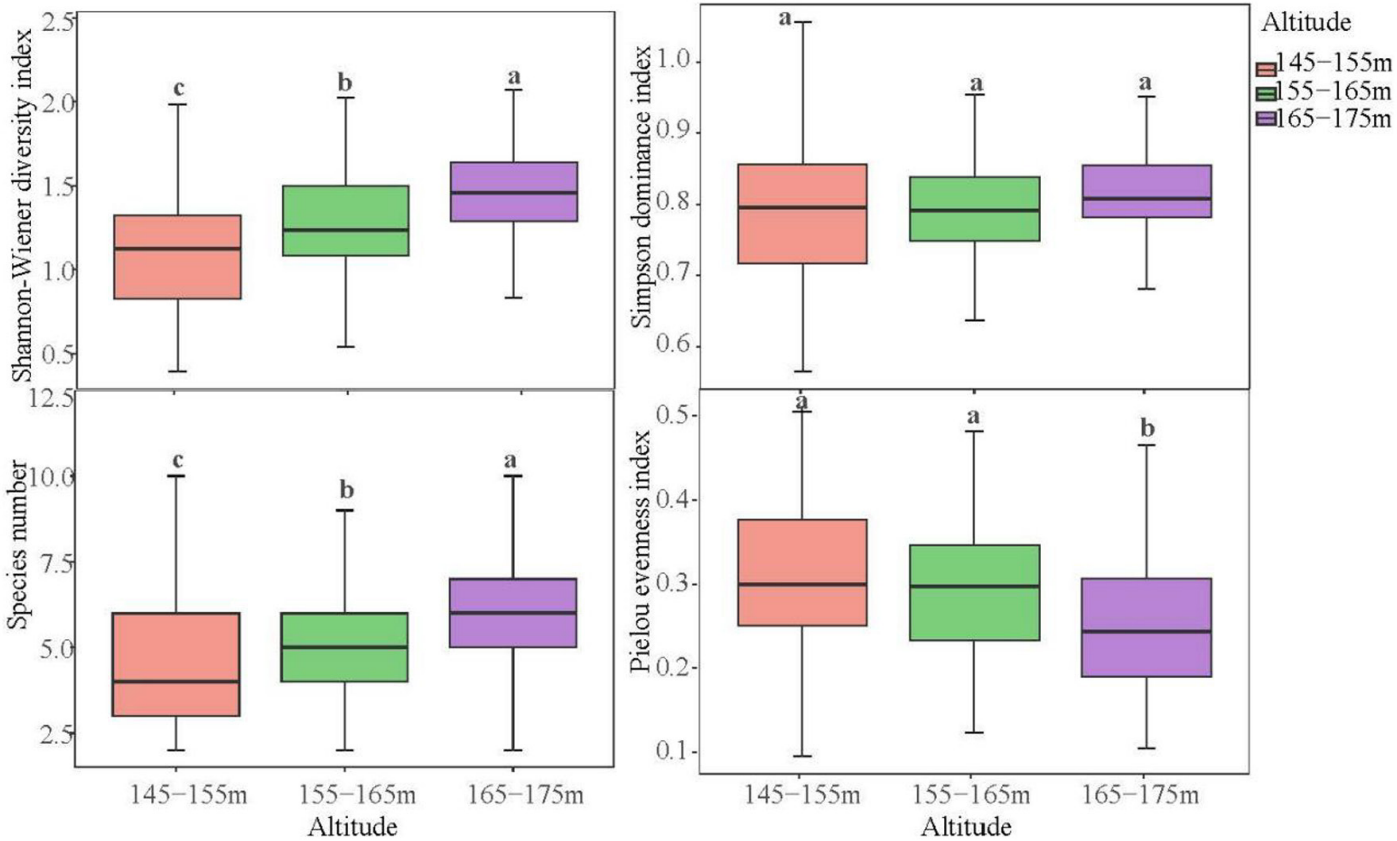
Plant diversity among different altitude areas in the HFZs of the TGR. The ends of the boxes represent the 25th and 75th percentiles, and the whiskers represent the 10th and 90th quartiles. Different letters indicate statistically significant differences among the three altitude areas at *p* < .05 (*n* = 105 for 145–155 m; *n* = 137 for 155–165 m; *n* = 126 for 165–175 m).

The Shannon diversity index (*H*) ranged from 0.831 to 1.661 in the HFZs, rising with the increasing altitude, of which the highest *H* (1.458 ± 0.035) appeared at the altitude of 165–175 m. The species number (*S*) and Simpson dominance index (*D*) also showed the same rising trend in spatial variation with altitude in the HFZs, but the latter is not significant. However, the Pielou evenness index (*E*) showed a contrary trend that *E* decreased with the increasing altitude (*p* < .01, Figure 3).

**FIGURE 4:**
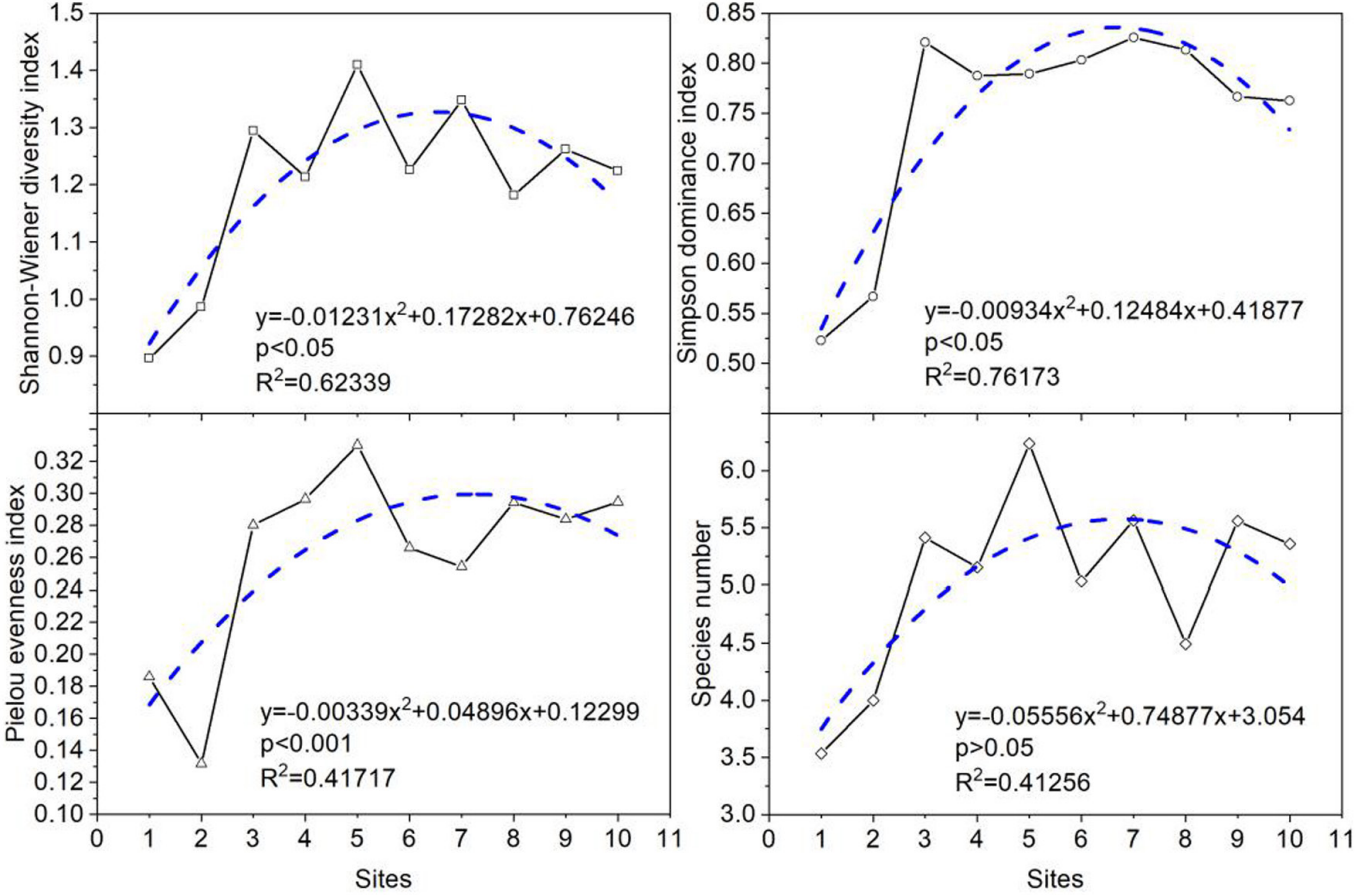
The spatial dynamics of plant diversities from upstream to downstream in the HFZs of the TGR. Regression analysis was used to investigate the spatial distribution pattern for each parameter and one‐way ANOVA was used to examine the differences among the different sampling sites. 1. Wujiang; 2. Zhongxian; 3. Wanzhou; 4. Pengxi River; 5. Tangxi River; 6. Meixi River; 7. Daxi River; 8. Daning River; 9. Xiangxi River; 10. Tongzhuang River.

The *H*, *D*, *S* and *E* showed same trends in the spatial distribution patterns from upstream to downstream in the HFZs of TGR, which firstly showed a significant increasing trend (*p* < .05), reached the highest in the middle reaches in the Tangxi River, and then decreased in the downstream reaches (Wujiang River to Tongzhuang River) (Figure 4). And the *H*, *E* and *S* reached the highest point at Tangxi River. In short, the spatial heterogeneity of the *H*, *D*, and *E* for plants from upstream to downstream was strong in the TGR of the Yangtze River but *E* showed a contrary trend.

### 3.3 Classification of guilds

**FIGURE 5:**
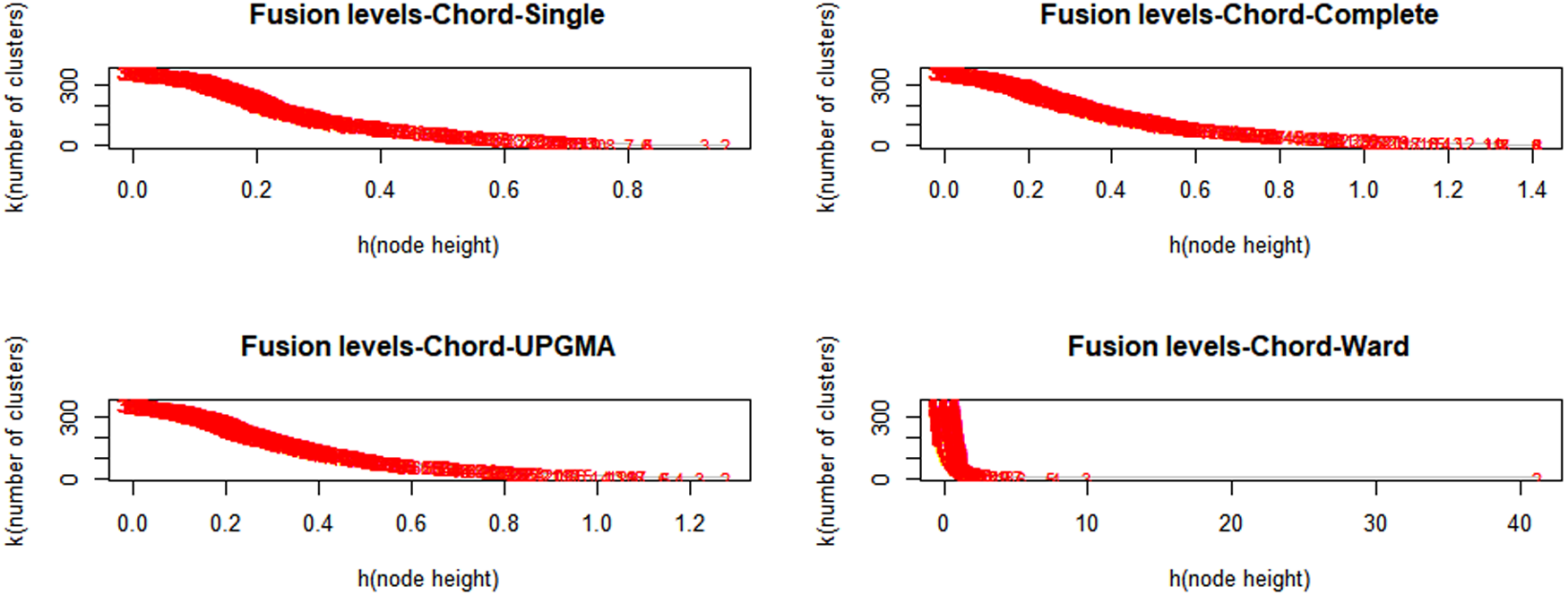
Fusion level diagram of four different clustering results.

In this study, the important values of species in the plant community were selected as the clustering basis. Four different clustering methods were used to cluster 368 quadrats (communities) in the HFZs (Figure 5). To interpret and compare the results of the four clustering analysis methods, we need to find the interpretable clusters. As the value of the difference between the two branches in the cluster tree, the fusion level value of the cluster tree can help to judge the clustering level of different clustering methods through analysis. The results of fusion level and clustering tree showed that the single link clustering was the most reasonable among the four clustering methods (Figure 5).

**FIGURE 6:**
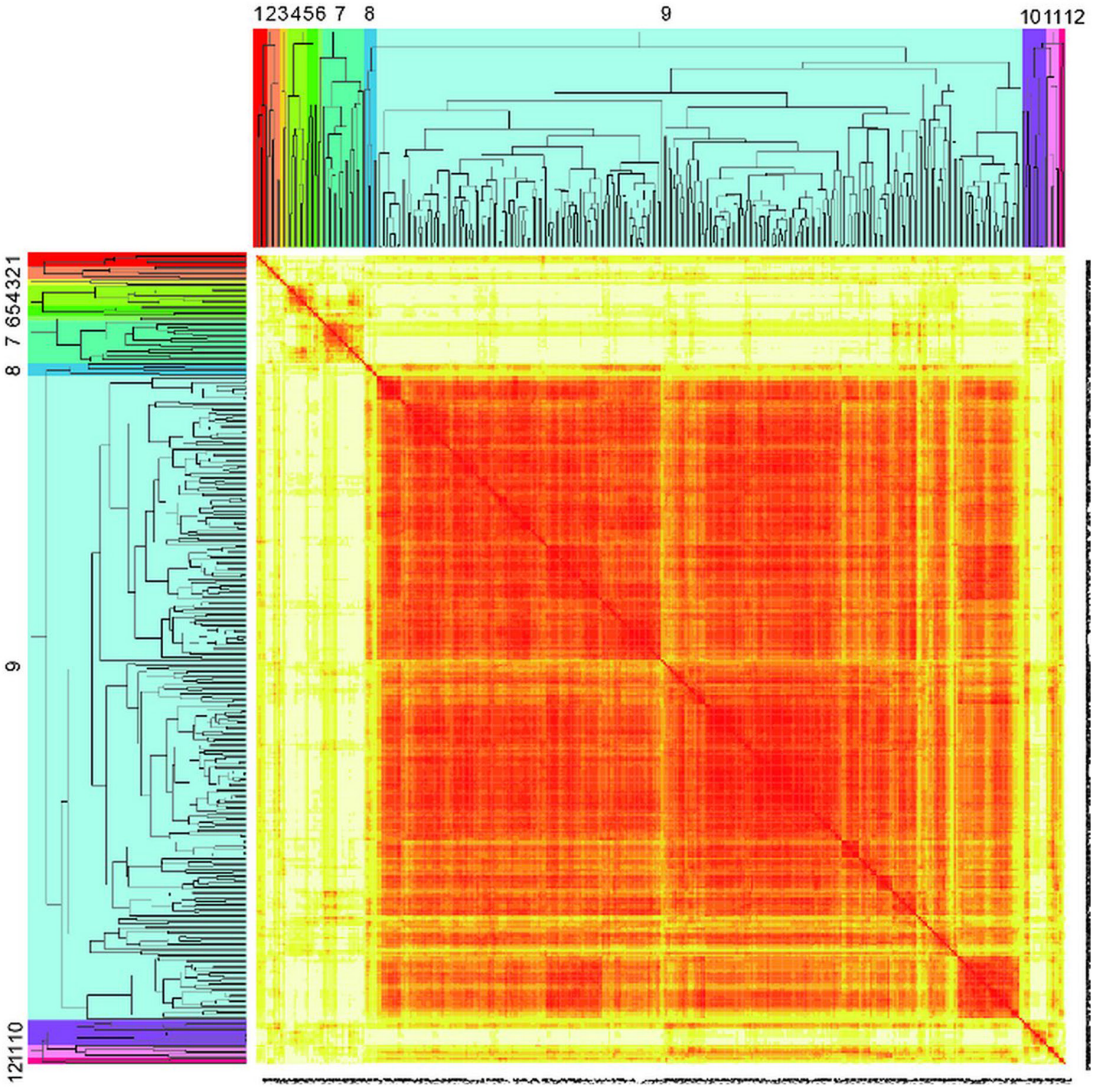
A heatmap of cluster tree of plant guilds on the distance matrix in the HFZs. The number on the figure represents the vegetation combination, refer to the Table S1 for details.

Then, the single chain clustering method was selected to cluster 368 samples in the HFZs of the TGR. By calculating the correlation between the original distance and the binary matrix representing different classification levels, the classification level corresponding to the highest correlation coefficient was selected as the optimal grouping scheme, and the results showed that it was the most reasonable to be divided into 12 guilds (Figure 6). Each guild represented a plant community type, and it was named after the dominant species in the community. The named format refers to the description of plant community name in 《Chinese Vegetation》 (Wu, 1980). The 12 main plant guilds were discovered as followed:

Guild 1. Ass. *Eclipta prostrata* + *Cynodon dactylon*, hygrophyte and mesophyte community, flood‐tolerant riparian herbs, including 5 samples and 16 species. The main companion species were *Digitaria sanguinalis*, *Euphorbia humifusa*, and *Acalypha australis*, with a distribution range of 155–175 m in elevation.

Guild 2. Ass. *Bidens pilosa*, hygrophyte and mesophyte community, competitive annual herbs, including 6 samples and 21 species. The companion species are *Polygonum hydropiper*, *Amaranthus retroflexus*, *Echinochloa crusgalli*, and *Xanthium sibiricum*, which were mainly found at elevations of 165–175 m.

Guild 3. Ass. *Bidens tripartita*, hygrophyte *and* mesophyte community, competitive annual herbs, including 3 samples and 13 species. The companion species are *Xanthium sibiricum*, *Digitaria sanguinalis*, *Echinochloa crusgalli*, *Bidens pilosa*, *Cynodon dactylon*, the main distribution altitude is 165–175 m.

Guild 4. Ass. *Digitaria sanguinalis*, hygrophyte *and* mesophyte community, competitive annual herbs, including 9 samples and 18 species. The companion species are *Setaria viridis*, *Acalypha australis*, and *Xanthium sibiricum*, which were mainly distributed at an elevation of 165–175 m.

Guild 5. Ass. *Echinochloa crusgalli* + *Digitaria sanguinalis* + *Setaria viridis*, mesophyte community, competitive riparian herbs, including 5 samples and 18 species. The companion species were *Bidens pilosa*, *Xanthium sibiricum* and *Humulus scandens*, which are mainly found at elevations of 145–175 m.

Guild 6. Ass. *Humulus scandens*, hygrophyte and mesophyte community, competitive riparian herbs, including 2 samples and 8 species. The companion species are *Bidens pilosa*, *Echinochloa crusgalli*, *Eleusine indica* and *Chenopodium ambrosioides* which were mainly found at elevations of 165–175 m.

Guild 7. Ass. *Setaria viridis*, hygrophyte and mesophyte community, competitive riparian herbs, including 19 samples and 28 species. The companion species are *Digitaria sanguinalis*, *Xanthium sibiricum*, *Bidens pilosa*, and *Cynodon dactylon* and *Eriochloa villosa*, which were mainly found at elevations of 165–175 m.

Guild 8. Ass. *Cynodon dactylon* + *Abutilon theophrasti* + *Salvia plebeia*, hygrophyte and mesophyte community, stress‐tolerant woody and herb species, including 6 samples and 15 species. The companion species are *Solanum nigrum*, *Digitaria sanguinalis*, *Bidens pilosa* and *Setaria viridis*, which were mainly found at elevations of 165–175 m.

Guild 9. Ass. *Cynodon dactylon*, hygrophyte and mesophyte community, flood‐tolerant riparian herbs, including 293 samples and 62 species. The companion species are *Xanthium sibiricum*, *Echinochloa crusgalli*, *Cyperus rotundus*, and *Bidens pilosa*, which were mainly found at elevations of 145–175 m.

Guild 10. Ass. *Conyza canadensis* + *Bidens pilosa*, hygrophyte and mesophyte community, competitive annual herbs, including 11 samples and 20 species. The companion species are *Artemisia argyi*, *Setaria viridis*, *Echinochloa crusgalli* and *Eriochloa villosa*, which were mainly found at elevations of 165–175 m.

Guild 11. Ass. *Cynodon dactylon* + *Melilotus officinalis*, hygrophyte and mesophyte community, stress‐tolerant herb and woody species, including 6 samples and 15 species. The companion species were *Setaria viridis*, *Anemarrhena asphodeloides*, *Echinochloa crusgalli*, *Bidens pilosa* and *Abutilon theophrasti*, which are mainly found at elevations of 155–175 m.

Guild 12. Ass. *Cynodon dactylon* + *Echinochloa crusgalli* + *Cyperus rotundus*, hygrophyte and mesophyte community, flood‐tolerant riparian herbs including 3 samples and 9 species. The companion species are *Amaranthus retroflexus*, *Xanthium sibiricum* and *Torulinium ferax*, which are mainly found at elevations of 145–155 m.

### 3.4 Response of plant communities in the TGR to water level changes and environmental factors

**FIGURE 7:**
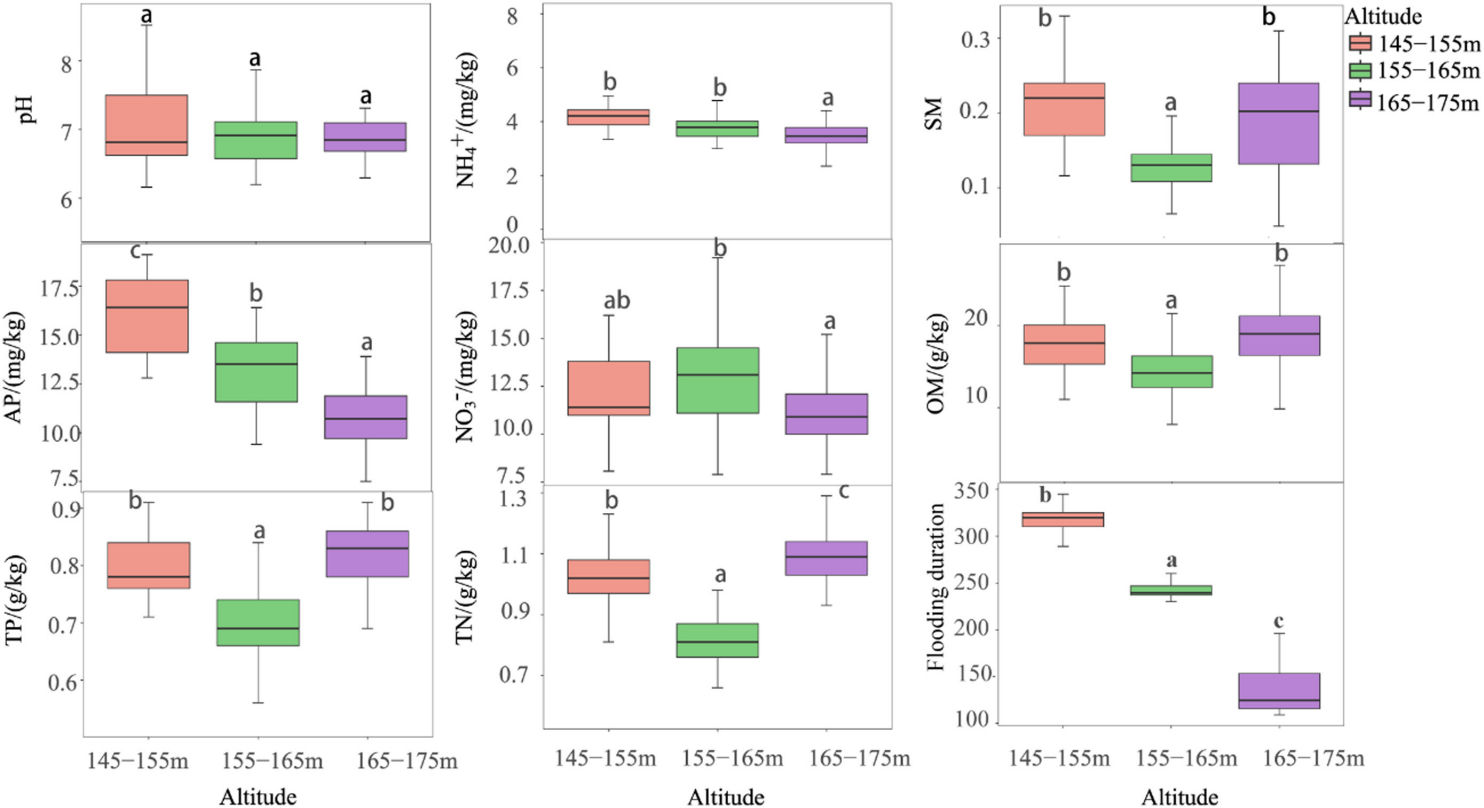
The heterogeneity of soil environmental factors in different habitats in the TGR. Low altitude, 145–155 m; Middle altitude, 155–165 m; High altitude, 165–175 m. The lower and upper edges of each box are 25th and 75th percentiles, and bars of each box indicate 10th and 90th percentiles. AP, available phosphorus; ${\mathrm{NH}}_{4}^{+}$, ammonium; ${\mathrm{NO}}_{3}^{-}$, nitrate; OM, organic matte; SM, soil moisture; TN, total nitrogen; TP, total phosphorus.

All soil chemistry properties were significant (*p* < .05) across sites (Figure 7). SM, OM, TN, TP and pH firstly decreased and then gradually increased with “V” type changes along the altitude gradient, of which the lowest in the TGR area at 155–165 m altitude, and all changes were significant except for pH. The AP, ${\mathrm{NH}}_{4}^{+}$, ${\mathrm{NO}}_{3}^{-}$ in the TGR area had a significant decreasing trend with the increasing altitude.

**FIGURE 8:**
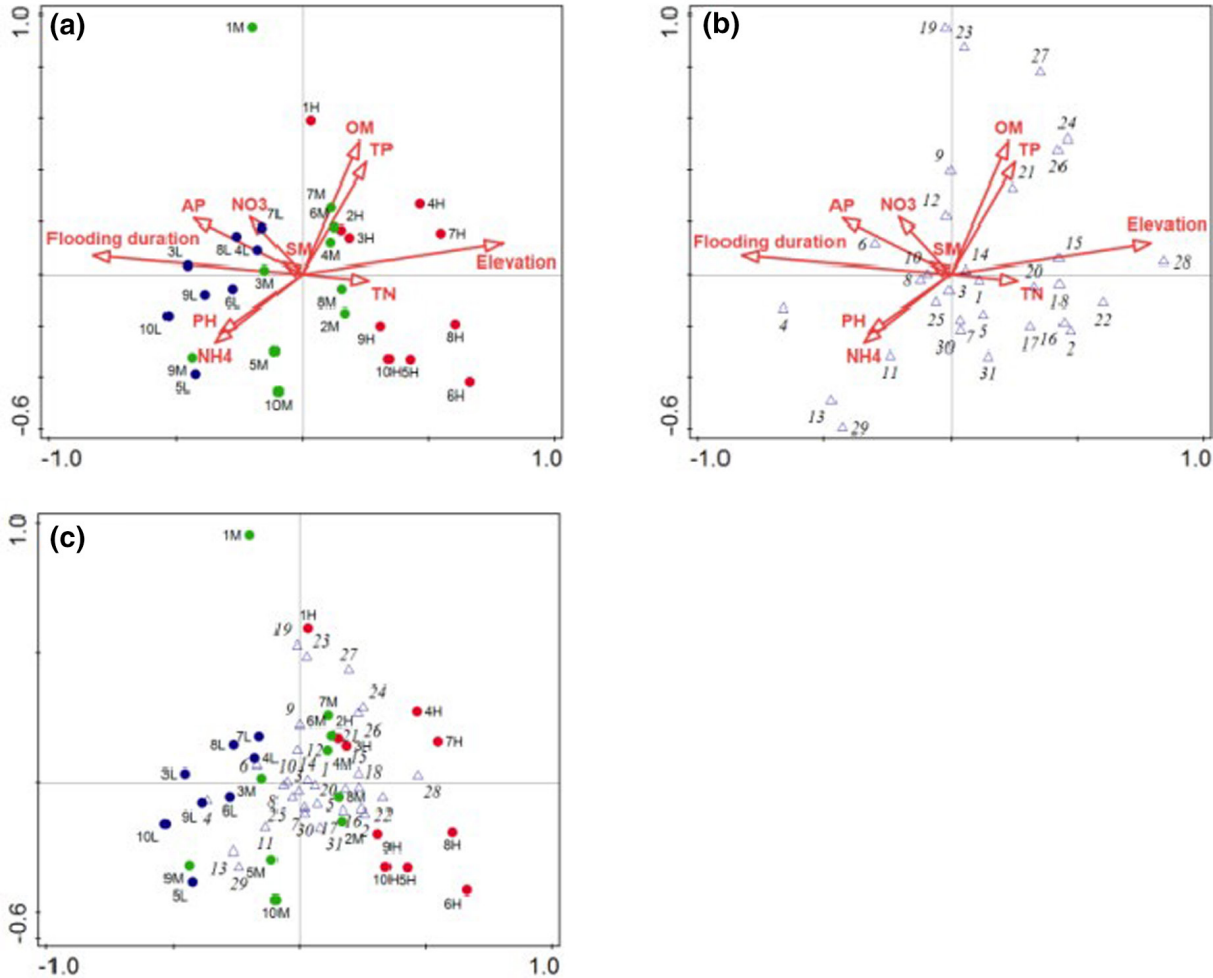
Ordination diagram showed the result of CCA analysis of the important value index of (a) sampling site distribution and environment variables, (b) species and environmental variables, and (c) sampling site distribution and plant species in the HFZs of the TGR. For a, b, and c, no. of sampling sites, environment variables and no. of plant species are presented in Tables S2–S4.

Soil environmental factors and hydrological factor in the HFZs significantly influenced the distribution pattern of plant guilds (Figure 8). The ranking of plant guilds in response to water level changes and environmental factors in the HFZs of the TGR is shown in Table 1, and the first four paradigmatic axes cumulatively explained 44.3% of the guild changes. The species‐environment correlation coefficients for axes 1 and 2 were 0.8615 and 0.8777, respectively, and the sum of the eigenvalues of the first two axes accounted for 62.83% of the total eigenvalues, containing most of the ranking information, so the data from the first two axes were used to analyze the relationship between guilds and environmental factors. As seen by the CCA ordination diagram (Figure 8), the position of each guild in the ordination space can reflect the ecological characteristics of the guilds and their distribution patterns. With the increasing altitude, the distribution patterns of the plant guilds in the WLFZ were like this. The lower altitude area (145–155 m) was dominated by annual plant guilds, i.e., *Echinochloa crusgalli and Amaranthus retroflexus*. The middle part (155–165 m) was dominated by annuals and perennial plant guilds, i.e., *Cynodon dactylon*, *Cyperus rotundus*, *Bidens pilosa*, *Xanthium sibiricum and Melilotus officinalis*. The top area (165–175 m) was dominated by shrub and herb plant guilds, i.e., *Echinochloa crusgalli*, *Cynodon dactylon*, *Eriochloa villosa* and *Abutilon theophrasti*.

**TABLE 1:** CCA ranking axis eigenvalues and interpretation.

	Axis 1	Axis 2	Axis 3	Axis 4
Eigenvalue	0.12	0.09	0.07	0.05
Explained changes (cumulative)	12.38	21.69	29.02	34.52
Species‐environmental correlation	0.86	0.88	0.80	0.81
Fitting variation in interpretation (cumulative)	27.96	48.99	65.55	77.97

### 3.5 Niche breadth

The niche breadth of species in the HFZs is listed in the Table S5, the top 8 of which were *C*. *dactylon* (21.918) > *X*. *sibiricum* (13.275) > *B*. *pilosa* (11.318) > *E*. *crusgalli* (9.472) > *C*. *rotundus* (6.407) > *Eclipta prostrata* (6.052) > *D*. *sanguinalis* (5.967) > *S*. *viridis* (5.816). And the niche breadth of *C*. *dactylon* was significantly larger than that of others. Under different altitude sections, *C*. *dactylon* (7.0836) > *E*. *crusgalli* (3.3973) > *B*. *pilosa* (3.1698) at the lower elevations (145–155 m); *C*. *dactylon* (8.176) > *X*. *sibiricum* (4.436) > *E*. *crusgalli* (3.721) at the middle elevations (155–165 m); *C*. *dactylon* (6.659) > *X*. *sibiricum* (6.0956) > *E*. *prostrata* (4.889) at the highest elevations (165–175 m). The dominant species had the different niche breadth in different elevations. Of these, the niche breadth of *C*. *dactylon* at the middle elevations (155–165 m) was the highest (8.176) and lowest at the higher elevations (165–175 m). And the niche breadth of *C*. *dactylon* was obvious higher than other species at the lower (145–155 m) and the middle elevations (155–165 m), but not at the highest elevations (165–175 m). The niche breadth of most of perennial herbs was higher at the highest elevations (165–175 m) than that at the lower (145–155 m) and middle elevations (155–165 m).

**FIGURE 9:**
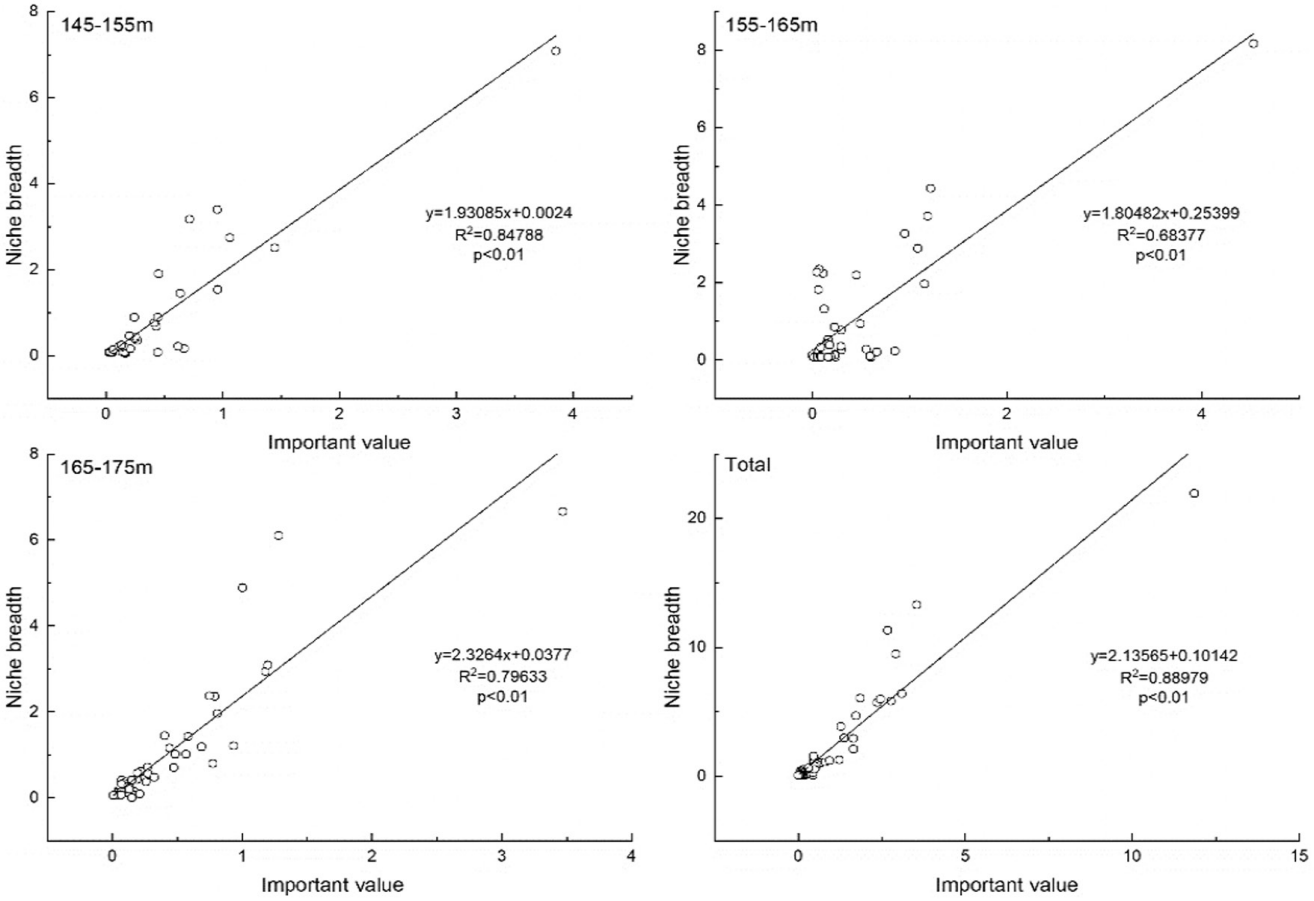
Correlation between niche breadth and important value in HFZs with elevations (145–155, 155–165, 165–175 m, and Total).

It could be seen from the correlation between niche breadths and important values of each elevation in the surveyed sample plot under three types of elevations were positively correlated (Figure 9). There was also a significant positive correlation between the niche breadths and the important values of each species, and the explanatory variables were high. The dominant species with high important value always had the higher niche breadths value. *C*. *dactylon* both had the highest niche breadths and important values.

### 3.6 Niche overlap

**FIGURE 10:**
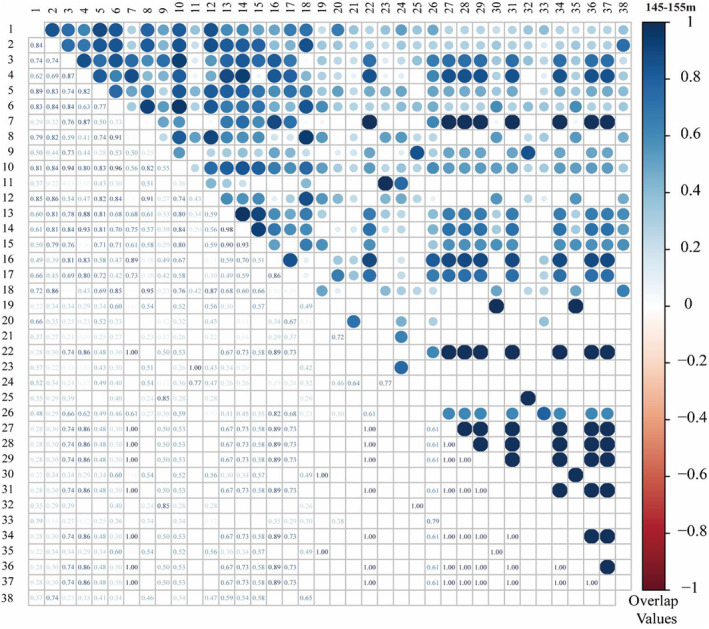
Niche overlap values for dominant herbaceous plants in the 145–155 m altitude section in the HFZs. No. (1–38): Plant species number (refer to Table S6 for complete plant species number).

Niche overlap by species for each altitude section was calculated. There were 703 niche overlap indexes calculated by 38 species at the altitude of 145–155 m, 33.85% of which were 0, 35.85% of which were greater than 0.5 and 38.55% of which were less than 0.2. *C*. *dactylon*, *X*. *sibiricum*, *S*. *viridis*, and *D*. *sanguinalis* all had the high niche overlap value compared with most of species. However, there were also some species with low niche breadths and important value, such as *H*. *scandens*, *L*. *crustacea* and *C*. *ambrosioides*, which had high niche overlap values (Figure 10).

There were 901 niches overlap indexes calculated by 43 species at the altitude of 155–165 m, 44.73% of which were 0, 18.09% of which were greater than 0.5 and 49.28% of which were less than 0.2. *C*. *dactylon*, *X*. *sibiricum*, *S*. *viridis*, and *D*. *sanguinalis* all had the high niche overlap value with most of species (Figure S1).

There were 1596 niches overlap indexes calculated by 57 species at the altitude of 165–175 m, 42.42% of which were 0, 20.86% of which were greater than 0.5 and 48.81% of which were less than 0.2. *C*. *dactylon*, *C*. *rotundus*, *X*. *sibiricum*, *P*. *hydropiper*, and *E*. *crusgalli* all had the high niche overlap value with most of species (Figure S2). Niche overlap index of the same species in different altitudes was different. The niche overlap values of *C*. *dactylon* were higher at the middle elevations (155–165 m) than that at the higher (165–175 m) and lower elevations (145–155 m).

## 4 DISCUSSION

### 4.1 Anti‐seasonal flooding substantially altered floristic composition and structures of plant communities in the HFZs of the TGR

A unique riparian ecosystem has been created as a result of anti‐seasonal and continuous flooding after TGR operations, which notably influenced the floristic composition of plant communities and their functional characteristics (Li et al., 2022). Before the impoundment of the TGR, there were 405 vascular plants in the riparian area of the TGR (Yong et al., 2002). However, in the early stage of the TGR impoundment in 2009, only a total of 231 species, belonging to 61 families and 169 genera, was found in the HFZs of the TGR (Liu et al., 2011). After the early 7 years of the TGR impoundment in 2010, Zhang et al. (2013) found that a few shrubs (*Boehmeria nivea*, *Lespedeza davidii*, *Lespedeza cuneata*) and trees (*Morus alba*, *Albizia kalkora* and *Broussonetia papyrifera*) only appeared at the altitude of 170 m. The main vegetation type was herbs in the HFZs. In the present study, after 19 years of the TGR impoundment, a total of 73 vascular plant species were identified in the HFZs. Annual herbs accounted for the highest percentage of all life forms at each altitude. Annuals, perennials and shrubs accounted for 71.23%, 27.39% and 1.37% of the total number of species, respectively. Thus it could be seen, anti‐seasonal and continuous flooding triggered the dramatic alterations in floristic composition and structure of plant communities in the riparian zone.

At the same time, after 19 years of water storage, plant life forms have been altered dramatically in the new riparian forest. This novel anti‐seasonal flooding reduced functional diversity, mostly owing to the loss of stress‐tolerant woody species and competitive perennial herbs as well as competitive annuals and flood‐tolerant riparian herbs, as the most abundant functional guilds, favored under such novel flooding conditions. Essentially new hydromorphological conditions following damming limited recruitment of native shrub and tree species guilds sensitive to floods (to drag forces, inundation, and anoxia). Thus it can be seen that woody plants (trees and shrubs) showed the greatest decrease, and the proportion of perennial herbs also decreased, whereas the proportion of annual herbs increased significantly, indicating that herbs, especially annual herbs, i.e. Compositae, Gramineae and Leguminosae families, were more suitable for the environment of water level fluctuations in the TGR. Thus it could be seen, the pre‐dam vegetation failed to persist under the new riparian ecosystem, and the species richness and diversity of the riparian forests were significantly lower due to the great hydrological shifts by the TGD construction (Chen et al., 2012, 2022; New & Xie, 2008).

A plausible reason was that most annuals, i.e., Compositae, Gramineae and Leguminosae, germinate in spring and fructify in autumn during the low water level of the TGR operation within a growth season, and have the flooding tolerance and the capacity to synchronize germination and growth within a short‐exposure period, which underlie the plant species alterations. Therefore, these adaptive annuals survive as dominant species in the HFZs of the TGR mainly because their phenology do not compound with the submergence occurring time. The recession of the water level left a nearly barren drawdown zone and provided an entire growing season for the forbs and graminoids, especially for the annual and biennial and perennial species. With its short life cycle, annual herbs were able to go from seed to seed life cycle in a relatively short period of time after water receding and before water storage. The next year, a new life cycle began with the emergence of seeds from nearby seed sources or soil seed banks (De Souza et al., 2021). After receding, clonal growth can quickly expand space and gain an advantage in the inter‐specific competition by their extensive lateral spread and forming dense, nearly monospecific stands, i.e., *C*. *dactylon*, which can quickly re‐sprout following continuous inundation and take advantage of the short‐exposure period before the reservoir is filled again. Thus, the transformed species richness of biennials and perennials increased significantly, especially the Compositae, Graminaceae and Leguminaceae plants.

After several times of water level fluctuations, structures of plant communities also altered in the riparian zones. The plant guilds distributed with the increasing elevation and decreasing flooding time, as followed: Ass. *C*. *dactylon* + *E*. *crusgalli* + *C*. *rotundus*; Ass. C. *dactylon* + *A*. *theophrasti* + *S*. *plebeia*; Ass. *E*. *crusgalli* + *D*. *sanguinalis* + *S*. *viridis*; Ass. *C*. *dactylon*; Ass. *B*. *pilosa*; Ass. *B*. *tripartita*; Ass. *D*. *sanguinalis*; Ass. *H*. *scandens*; Ass. *S*. *viridis*; Ass. *C*. *canadensis* + *B*. *pilosa*; Ass. *E*. *prostrata* + *C*. *dactylon*; Ass. *C*. *dactylon* + *M*. *officinalis* (Figure 8a). There were some overlaps between the types, Ass. *C*. *dactylon* + *E*. *crusgalli* + *C*. *rotundus* and Ass. C. *dactylon* + *A*. *theophrasti* + *S*. *plebeia* located at the altitude gradient of 145–155 m, with long flooding durations and short growth durations. The medium elevation gradient (155–165 m) was for the Ass. *E*. *crusgalli* + *D*. *sanguinalis* + *S*. *viridis* and Ass. *C*. *dactylon*. Ass.*B*. *pilosa*, Ass. *B*. *tripartita*, Ass. *D*. *sanguinalis*, Ass. *H*. *scandens*, Ass. *S*. *viridis*, Ass. *C*. *canadensis* + *B*. *pilosa*, Ass. *E*. *prostrata* + *C*. *dactylon* and Ass. *C*. *dactylon* + *M*. *officinalis* were distributed at high altitudes (165–175 m) in the HFZs, with less or almost unaffected by flooding. In the early stages of flooding in 2010, there were 18 main plant guilds in the HFZs of the TGR, including 5 xerophyte, 6 hygrophyte and 7 mesophyte guilds (Chen et al., 2012). In the present study, after 19 times of water level fluctuations in the TGR, the 12 main plant guild types were discovered, belonging to hygrophyte and mesophyte communities. Xerophyte guilds almost disappeared. Thus, the taxonomic and functional characteristics of communities were differently as they may respond to important drivers differently. The vegetation composition of the HFZs in the TGR showed a **s**ignificant change with a transition from xerophytes to hygrophytes and mesophytes with the increasing flooding time. The observed riparian plant guild response patterns to prolonged submergence in the HFZs of Yangtze River might hopefully be transferred to similar rivers with little or no existing information in other regions regardless of whether or not they share species. The guild approach helps develop general frameworks to predict vegetation responses to changing environmental conditions.

### 4.2 Anti‐seasonal flooding substantially altered species diversity distribution patterns of plant communities in the in the HFZs of the TGR

Species diversity distribution along elevation gradients has different patterns. Some studies have demonstrated that species richness patterns from the lowest to highest elevations may show a monotonic decrease, or a monotonic increase; others have revealed hump‐shaped patterns with a peak in richness at mid‐elevations (Mallen‐Cooper & Pickering, 2008; Trigas et al., 2013). In the present study, the S, H and D increased with the increasing elevation but E showed a contrary trend (Figure 3). This species distribution pattern might be caused by several synergetic attributes (e.g., the submergence depth, the tolerant capacity of plants to flooding, the life form, the dispersal mode, and the inter‐specific competition) (Zhang et al., 2013). The lower elevation area (145–155 m) is generally prone to more severe flooding with a greatest depth of inundation (30 m) and prolonged inundation duration (nearly half 6 months), which resulted in the lower S, and H than that in the higher elevation area (155–165 and 165–175 m). However, H showed hump‐shaped patterns with a peak at mid‐reaches from upstream to downstream (Figure 4). The middle reaches of the reservoir area were mostly forested on both sides of the Yangtze River, with less agricultural cultivation and less human influence. In the downstream reaches, the Citrus farming industry on both sides of the Xiangxi and Tongzhuang rivers was booming, and the plant communities on both sides of the river was more affected by agricultural farming and human interference. Ecological characteristics of plant guilds as an assemblage of plant population are response to the environment changes and are more pronounced during succession (Ge et al., 2020; Yang, Chen, et al., 2012; Yang, Liu, et al., 2012). In this study, it was found that the main influence factors affecting the spatial distribution of the plant guilds in the new riparian forest were hydrological factors, such as elevation (different flooding depths) and flooding time. So, the patterns of the plant guilds in the TGR were mainly affected by water level disturbance. This result was largely consistent with other results that hydrological conditions determined the vegetation diversity and aboveground biomass patterns at the elevation gradients of the drawdown zone (Wang et al., 2014). It could be seen that the vegetation spatial distribution of the TGR area was heavily influenced by the hydrological factors, i.e., different flooding depth and flooding time in the reservoir area and the species diversity was significantly reduced.

Soil nutrient concentrations could also strongly affect plant species diversity and evenness (Veen et al., 2024). In the present study, the concentrations of TN and TP appeared to be higher at the elevations of 165–175 m than at the elevations of 145–155 and 155–165 m. The continuous submergence in winter and the high frequency of floods in summer may result in this pattern for soil nutrients may be released when submerged and soil that serves as a nutrient source may be scoured by repeated flooding. In addition, soil environmental factors and hydrological factor in the HFZs significantly influenced the distribution pattern of plant guilds (Figure 8). In particular, the correlation between flooded time and elevation with axis 1 was the highest (0.8297 and 0.7943, respectively). Meanwhile, the correlation between OM and TP with axis 2 was notably higher (0.5119 and 0.4327, respectively). These findings are congruent with the research conducted by Yang, Liu, et al. (2012), Yang, Chen, et al. (2012), and Yang, Liu, et al. (2012), wherein they documented a reduction in TN concentration subsequent to prolonged inundation. And Roem and Berendse (2000) studied nutrient supply ratio as possible factors determining changes in plant species diversity in grassland and heathland communities, which showed that plant species with high diversity were at balanced N/P ratios between 10 and 14. However, in the present study, N/P ratios of the soil were between 0.98 and 1.56 with an average of 1.26, which showed that N was a limiting factor in the soil in the new riparian forest. The increase of N supply in a N‐limited grassland (e.g., N/P ratio < 10) may lead to an increase in biodiversity (Roem & Berendse, 2000). In this study, the distribution patterns of plant guilds were positively associated with TN, TP, and OM, whereas negatively correlated with pH, ${\mathrm{NH}}_{4}^{+}‐N$, ${\mathrm{NO}}_{3}^{-}‐N$, and AP in the CCA (Figure 8b). Of these, the significantly negative correlation between ${\mathrm{NO}}_{3}^{-}‐N$ and the distribution patterns of plant species (*p* < .05) was consistent with the results that excess of ${\mathrm{NO}}_{3}^{-}‐N$ is known for its negative effect on the diversity of plant guilds (Aerts et al., 2003). Therefore, N might be a soil nutrient limiting factor in determining the alterations in plant species diversity and plant distribution patterns in addition to elevation gradients and flooding time in the new riparian forest of the TGR.

The results also corroborated the trend of the species diversity index increasing with the elevation gradients. At higher elevations, the number of plant species is increasing and the competition between species becoming more intense. From bottom to top along axis 2, OM and TP rose (Figure 8b). This result is basically the same as the pattern shown in Table 2, which showed that the influence of elevation and flooding on the distribution pattern of the plant guilds in the TGR was more obvious than that of OM and TP, and the first order axis could explain the interrelationship between the plant guild and the habitat in the declining zone (Figure 8c). That is, although the spatial distribution of the plant guilds in the subduction zone of the TGR was the result of a combination of multiple factors, the influence of elevation gradients and flooding time played a dominant role in the formation of the spatial pattern of the plant guilds in the new riparian forest of the TGR and the second factors were TN, TP, and OM.

**TABLE 2:** Correlation of 10 soil chemistry properties with each CCA axis.

	Axis 1	Axis 2	Axis 3	Axis 4
Elevation	0.7943	0.1183	0.0124	0.0287
Flooding duration	−0.8297	0.0702	0.0519	−0.0828
SM	−0.0779	0.0455	0.2981	−0.7346
AP	−0.4317	0.2144	0.1826	−0.1407
${\mathrm{NH}}_{4}^{+}$	−0.3453	−0.2666	0.1734	−0.0061
${\mathrm{NO}}_{3}^{-}$	−0.2049	0.2193	0.3508	0.0255
OM	0.2296	0.5119	−0.3025	0.0315
pH	−0.3274	−0.2285	−0.2558	0.2327
TN	0.2662	−0.0272	0.0923	−0.3163
TP	0.2579	0.4327	0.5212	−0.1251

### 4.3 Niche structure and utilization of limited resources

Niche breadth describes a suite of environments or resources, in the broadest sense, which a species can inhabit or use, which measures the range of resource characteristics across which a species exists, and indicates the extent that a species utilizes different types of resources (Slatyer et al., 2013), whereas niche overlap refers to the partial or complete sharing of resources or other ecological factors (predators, foraging space, soil type, and so on) by two or more species (Colwell & Futuyma, 1971). The measures of niche breadth and overlap are all based on the distribution of individual organisms, by species, within a set of resource states (Colwell & Futuyma, 1971). In the present study, the dominant species were *C*. *dactylon*, *X*. *sibiricum*, *C*. *rotundus*, *E*. *crusgalli*, *S*. *viridis*, *B*. *pilosa*, *D*. *sanguinalis* and *P*. *hydropiper* in the riparian forest of the TGR according to the importance value and niche breadth. The correlations between niche breadths and important values of each elevation were positively correlated in the surveyed sample plots under three types of elevations (Figure 3). Under different altitude sections according to niche breadth, *C*. *dactylon* (7.0836) > *E*. *crusgalli* (3.3973) > *B*. *pilosa* (3.1698) at the lower elevations (145–155 m); *C*. *dactylon* (8.176) > *X*. *sibiricum* (4.436) > *E*. *crusgalli* (3.721) at the middle elevations (155–165 m); *C*. *dactylon* (6.659) > *X*. *sibiricum* (6.0956) > *E*. *prostrata* (4.889) at the highest elevations (165–175 m). *C*. *dactylon* was the most dominant species in the novel riparian forest with highest importance value and niche breadth at each altitude. *C*. *dactylon* might have evolved morphological, physiological, and biochemical adaptations to oxygen deficiency, such as dormant tubers or rhizomes (Zhang et al., 2013). So, it could germinate quickly after submerged period to go against the coming dry period to achieve the greatest competitive advantages in the HFZs. There were the most of species pairs with less than 0.2 or 0 of the niche overlap index at the middle altitude (155–165 m). The vegetation in the middle altitude (155–165 m) was the least affected by the Yangtze River flooding during the dry period. *C*. *dactylon* almost formed a single‐species community at the middle altitude (155–165 m), because of its strong acclimation and fast growth as well as facile vegetative propagation compared with other species.

Niche breadth in the study area had high niche overlap between species, but in some habitat conditions, the species with narrower niche breadth appeared larger niche overlap. There were some plants, such as *H*. *scandens*, *L*. *crustacea* and *C*. *ambrosioides*, niche overlap value was 1.00, almost perfect overlap. Most of these species had lower niche overlap with those dominant species. In fact, the niche occupation of resource space between two species was only infinitely close, so the overlap was only infinitely close to 1.00. This indicated that there was no direct linear relationship between niche breadth and niche overlap, which was caused by the heterogeneity of spatial distribution of environmental resources available to species (Chen et al., 2019). The *TAO*_*ih*_ in the different elevation areas was highest at the altitude of 145–155 m (0.3642), lower at 165–175 m (0. 2619) and 155–165 m (0.2524) in descending order. The vegetation in the lower elevations (145–155 m) suffered the longest periods of winter flooding and summer flood. So they had the shortest time to complete their life cycle under the influence of both winter storage and summer flood. The results showed that anti‐seasonal and continuous flooding would lead to the gradual disappearance of the original diverse niches, resulting in more uniform habitats, and more obvious competition among species with similar resource requirements. The comprehensive niche analysis concluded that, after 19‐year inundation of the TGR, the vegetation of the new riparian forest was still in the high niche overlap, intense competition, and species specialization, which showed that the vegetation was still in the early stage of primary succession, ecosystem stability was poor, and habitat fragmentation was severe in the TGR area.

### 4.4 Practical implications for vegetation restoration and reconstruction

The anti‐seasonal and continuous flooding precipitated loss of the original vegetation, especially trees and shrubs after the filling of the TGR. We found a significant decrease in the number of vascular plants compared to the pre‐flooding period. The proportion of annual herbs, especially Compositae, Gramineae and Leguminosae plants significantly increased in the riparian forest as a result of their adaptation strategies. And we found some invasive plants such as *E*. *annuus* began to show dominance in the vegetation composition of the new riparian forest in the TGR. The TGR area is not only one of the most biodiverse areas in China but also one of the most endemic species areas in the world (Jin et al., 1984). The dominance of invasive plants can cause great harm to the gene pool and genetic diversity in the TGR area (Ge et al., 2020; Yang, Chen, et al., 2012; Yang, Liu, et al., 2012). The present results showed the high heterogeneity of species diversity and environmental factors in the TGR areas with significant habitat changes and poor ecosystem stability. Therefore, there may be some differences in the governance strategies adopted in different areas of the novel riparian ecosystem for vegetation restoration of the riparian forests.

According to the comparative analysis of the vegetation status in the TGR area, the following four implications are proposed:

1. More attentions should be given to the indigenous species in the selection of species for the restoration and reconstruction of the novel riparian forests. The exploration and study of indigenous species in the reservoir may be a more effective and safe means of artificial vegetation restoration. Therefore, the above‐ described indigenous plant guilds should be prioritized in vegetation restoration efforts.
2. Differences between regions should be taken into account in the implement of vegetation restoration measures in the reservoir area. The construction of artificial guilds during vegetation restoration should be tailored to the local context and plant adaptations to local conditions.
3. In general, when the artificial guilds restored in the reservoir area, it may have a better effect with herbaceous plants as the main part, supplemented by shrubs or small trees in the middle‐high elevation areas such as *Distylium chinense* (Sun et al., 2020) and *Taxodium distichum* (Li et al., 2010).
4. Studies showed that stabilization of vegetation in the depression zone may take 70 years or more (Nilsson et al., 2013, 2015; Nilsson & Aradóttir, 2013). Although it has been 19 years since the formation of the HFZs of the TGR, the niche differentiation between different dominant plants was lower, the inter‐specific competition was more intense and the stability of plant guilds was still worse. Therefore, long term investigations and observations should be continued in this area to identify and monitor alterations in the characteristics of the plant guilds and soil properties due to anti‐seasonal and continuous inundation on riparian areas triggered by flow regulation or global warmer climates.

## 5 CONCLUSIONS

In the present study, the results revealed the diversity and niche characteristics of plant communities in the HFZs of the TGR and emphasized the significant negative effects of anti‐seasonal water level alteration on plant diversity and species competition, as well as the ecological characteristics in the early stage of vegetation succession–low diversity, high niche overlap and single species dominance. Studies have shown that hydrological regulation is a key factor affecting vegetation distribution, exceeding the effects of soil nutrients such as TN, TP, and OM. In addition, annual herbs plants showed better environmental adaptability, suggesting that vegetation restoration strategies should be biased towards herbaceous plants, supplemented by shrubs and small trees. To establish a complete reference system for vegetation restoration, natural vegetation monitory plots in the different succession stages should be established in the different HFZs of the TGR, and their environmental conditions, community structures and inter‐specific relationships analyzed.

## AUTHOR CONTRIBUTIONS

**Xiaoling Li:** Conceptualization (equal); formal analysis (equal); investigation (equal); supervision (equal); writing – original draft (equal); writing – review and editing (equal). **Ye Liu:** Investigation (equal); writing‐original (equal). **Wenxiong Yi:** Investigation (equal); writing – original draft (equal). **Xiaodie Duan:** Investigation (equal); writing – original draft (equal). **Gong Chen:** Investigation (equal); methodology (equal); software (equal). **Jin Yang:** Investigation (equal); methodology (equal); software (equal). **Danli Deng:** Funding acquisition (equal); project administration (equal); resources (equal); supervision (equal). **Xiaojuan Guo:** Funding acquisition (equal); writing – review and editing (equal). **Zhengjian Yang:** Funding acquisition (equal); writing – review and editing (equal). **Guiyun Huang:** Funding acquisition (equal); writing – review and editing (equal). **Meixiang Hu:** Funding acquisition (equal); writing – review and editing (equal). **Chen Ye:** Funding acquisition (equal); project administration (equal); resources (equal); supervision (equal).

## CONFLICT OF INTEREST STATEMENT

The authors declare that they have no known competing financial, interests or personal relationships that could have appeared to influence the work reported in this paper.

### OPEN RESEARCH BADGES

This article has earned an Open Data badge for making publicly available the digitally‐shareable data necessary to reproduce the reported results. The data is available at Appendix S1.

## Supporting information


Appendix S1.

## Data Availability

The authors confirm that the data supporting the findings of this study are available within the article. Raw data that support the findings of this study area available from the corresponding author upon responsible request.
