# Supplementary material for: Anti‐seasonal flooding drives substantial alterations in riparian plant diversity and niche characteristics in a unique hydro‐fluctuation zone
Source: Ecol Evol. 2024 Aug 9;14(8):e70036. doi: 10.1002/ece3.70036 (PMC11310770; doi:10.1002/ece3.70036)
Supplement: Supplementary file 1 — Appendix S1. [file ECE3-14-e70036-s001.docx]

**Supporting information**

**Table** **S1** Detailed results of the 12 plant guilds

| Number | Plant guilds |
| --- | --- |
| 1 | Ass. *Eclipta prostrata* + *Cynodon dactylon* |
| 2 | Ass. *Bidens pilosa* |
| 3 | Ass. *Bidens tripartita* |
| 4 | Ass. *Digitaria sanguinalis* |
| 5 | Ass. *Echinochloa crusgalli* + *Digitaria sanguinalis* + *Setaria viridis* |
| 6 | Ass. *Humulus scandens* |
| 7 | Ass. *Setaria viridis* |
| 8 | Ass. *Cynodon dactylon* + *Abutilon theophrasti* + *Salvia plebeia* |
| 9 | Ass. *Cynodon dactylon* |
| 10 | Ass. *Conyza canadensis* + *Bidens pilosa* |
| 11 | Ass. *Cynodon dactylon* + *Melilotus officinalis* |
| 12 | Ass. *Cynodon dactylon* + *Echinochloa crusgalli* + *Cyperus rotundus* |

**Table S2** Details of Figure 8. Abbreviations and full names of different soil environmental variables

| Variables | Abbreviation |
| --- | --- |
| pH | pH |
| Soil Moisture | SM |
| Organic Matter | OM |
| Total Nitrogen | TN |
| Nitrate | NO_3_^-^ |
| Ammonium | NH_4_^+^ |
| Total Phosphorus | TP |
| Available Phosphorus | AP |
| Total Potassium | TK |
| Available Potassium | AK |

**Table** **S3** Details of Figure 8a. The numbers used in the figure represent different sampling site names. L,M and H represent different elevations of 145-155m, 155-165m, and 165-175m.

| Number | Sampling site | Elevation (m) | Shape |
| --- | --- | --- | --- |
| 1 | Wujiang | L: Low elevation,145-155 | Circles |
| 1 | Wujiang | M: Middle elevation, 155-165 | Circles |
| 1 | Wujiang | H: High elevation, 165-175 | Circles |
| 2 | Zhongxian | L: Low elevation, 145-155 | Circles |
| 2 | Zhongxian | M: Middle elevation, 155-165 | Circles |
| 2 | Zhongxian | H: High elevation, 165-175 | Circles |
| 3 | Wanzhou | L: Low elevation,145-155 | Circles |
| 3 | Wanzhou | M: Middle elevation, 155-165 | Circles |
| 3 | Wanzhou | H: High elevation,165-175 | Circles |
| 4 | Pengxi River | L: Low elevation, 145-155 | Circles |
| 4 | Pengxi River | M: Middle elevation, 155-165 | Circles |
| 4 | Pengxi River | H: High elevation,165-175 | Circles |
| 5 | Tangxi River | L: Low elevation, 145-155 | Circles |
| 5 | Tangxi River | M: Middle elevation, 155-165 | Circles |
| 5 | Tangxi River | H: High elevation,165-175 | Circles |
| 6 | Meixi River | L: Low elevation,145-155 | Circles |
| 6 | Meixi River | M: Middle elevation, 155-165 | Circles |
| 6 | Meixi River | H: High elevation, 165-175 | Circles |
| 7 | Daxi River | L: Low elevation, 145-155 | Circles |
| 7 | Daxi River | M: Middle elevation, 155-165 | Circles |
| 7 | Daxi River | H: High elevation, 165-175 | Circles |
| 8 | Daning River | L: Low elevation,145-155 | Circles |
| 8 | Daning River | M: Middle elevation, 155-165 | Circles |
| 8 | Daning River | H: High elevation, 165-175 | Circles |
| 9 | Xiangxi River | L: Low elevation, 145-155 | Circles |
| 9 | Xiangxi River | M: Middle elevation, 155-165 | Circles |
| 9 | Xiangxi River | H: High elevation, 165-175 | Circles |
| 10 | Tongzhuang River | L: Low elevation, 145-155 | Circles |
| 10 | Tongzhuang River | M: Middle elevation, 155-165 | Circles |
| 10 | Tongzhuang River | H: High elevation, 165-175 | Circles |

**Table** **S4** Details of Figure 8b. The numbers used in the figure represent different plant species.

| Number | Plant species | Shape |
| --- | --- | --- |
| 1 | *Xanthium sibiricum* | Triangles |
| 2 | *Amaranthus retroflexus* | Triangles |
| 3 | *Bidens pilosa* | Triangles |
| 4 | *Portulaca oleracea* | Triangles |
| 5 | *Acalypha australis* | Triangles |
| 6 | *Cyperus rotundus* | Triangles |
| 7 | *Eclipta prostrata* | Triangles |
| 8 | *Echinochloa crusgalli* | Triangles |
| 9 | *Eleusine indica* | Triangles |
| 10 | *Cynodon dactylon* | Triangles |
| 11 | *Abutilon theophrasti* | Triangles |
| 12 | *Polygonum hydropiper* | Triangles |
| 13 | *Phyllanthus urinaria* | Triangles |
| 14 | *Solanum nigrum* | Triangles |
| 15 | *Digitaria sanguinalis* | Triangles |
| 16 | *Setaria viridis* | Triangles |
| 17 | *Eriochloa villosa* | Triangles |
| 18 | *Ageratum conyzoides* | Triangles |
| 19 | *Alternanthera philoxeroides* | Triangles |
| 20 | *Bidens tripartita* | Triangles |
| 21 | *Humulus scandens* | Triangles |
| 22 | *Melilotus officinalis* | Triangles |
| 23 | *Artemisia argyi* | Triangles |
| 24 | *Erigeron annuus* | Triangles |
| 25 | *Cyperus difformis* | Triangles |
| 26 | *Aster tataricus* | Triangles |
| 27 | *Artemisia carvifolia* | Triangles |
| 28 | *Pouzolzia zeylanica* | Triangles |
| 29 | *Salvia plebeia* | Triangles |
| 30 | *Aeschynomene indica* | Triangles |
| 31 | *Euphorbia humifusa* | Triangles |

**Table S5** Importance values and niche breadth of dominant plants at different altitudes in the HFZs

| Species | P_i_ | | | | B_i_ | | | |
| --- | --- | --- | --- | --- | --- | --- | --- | --- |
|  | 145-155 m | 155-165 m | 165-175 m | Total | 145-155 m | 155-165 m | 165-175 m | Total |
| *Cynodon dactylon* | 3.854 | 4.537 | 3.470 | 11.861 | 7.084 | 8.176 | 6.659 | 21.918 |
| *Xanthium sibiricum* | 1.061 | 1.215 | 1.280 | 3.556 | 2.743 | 4.436 | 6.096 | 13.275 |
| *Cyperus rotundus* | 1.444 | 1.085 | 0.567 | 3.097 | 2.505 | 2.886 | 1.016 | 6.407 |
| *Echinochloa crusgalli* | 0.956 | 1.183 | 0.787 | 2.926 | 3.397 | 3.721 | 2.353 | 9.472 |
| *Setaria viridis* | 0.415 | 1.157 | 1.199 | 2.772 | 0.766 | 1.960 | 3.091 | 5.816 |
| *Bidens pilosa* | 0.717 | 0.951 | 1.003 | 2.672 | 3.170 | 3.259 | 4.889 | 11.318 |
| *Digitaria sanguinalis* | 0.433 | 0.848 | 1.179 | 2.460 | 0.679 | 2.345 | 2.943 | 5.967 |
| *Polygonum hydropiper* | 0.957 | 0.665 | 0.747 | 2.369 | 1.536 | 1.811 | 2.365 | 5.712 |
| *Eclipta prostrata* | 0.454 | 0.592 | 0.809 | 1.856 | 1.901 | 2.188 | 1.963 | 6.052 |
| *Abutilon theophrasti* | 0.636 | 0.604 | 0.486 | 1.726 | 1.447 | 2.236 | 1.015 | 4.698 |
| *Alternanthera philoxeroides* | 0.671 | 0.300 | 0.689 | 1.659 | 0.154 | 0.766 | 1.183 | 2.103 |
| *Bidens tripartita* | 0.621 | 0.453 | 0.581 | 1.655 | 0.216 | 1.314 | 1.417 | 2.947 |
| *Acalypha australis* | 0.445 | 0.494 | 0.438 | 1.377 | 0.893 | 0.936 | 1.153 | 2.981 |
| *Solanum nigrum* | 0.243 | 0.555 | 0.473 | 1.271 | 0.885 | 2.271 | 0.707 | 3.863 |
| *Eriochloa villosa* | 0.151 | 0.300 | 0.773 | 1.224 | 0.214 | 0.253 | 0.796 | 1.263 |
| *Melilotus officinalis* | - | - | 0.937 | 0.937 | - | - | 1.210 | 1.210 |
| *Eleusine indica* | 0.249 | 0.234 | 0.325 | 0.808 | 0.394 | 0.222 | 0.479 | 1.096 |
| *Cyperus difformis* | 0.270 | 0.176 | 0.221 | 0.667 | 0.353 | 0.067 | 0.620 | 1.039 |
| *Humulus scandens* | 0.129 | 0.231 | 0.190 | 0.550 | 0.175 | 0.326 | 0.425 | 0.926 |
| *Artemisia argyi* | 0.164 | 0.162 | 0.196 | 0.521 | 0.077 | 0.067 | 0.570 | 0.713 |
| *Ageratum conyzoides* | 0.033 | 0.189 | 0.259 | 0.482 | 0.077 | 0.079 | 0.369 | 0.525 |
| *Euphorbia humifusa* | 0.035 | 0.294 | 0.151 | 0.480 | 0.077 | 0.348 | 0.407 | 0.832 |
| *Aeschynomene indica* | 0.160 | 0.178 | 0.134 | 0.472 | 0.083 | 0.382 | 0.198 | 0.664 |
| *Conyza canadensis* | - | 0.057 | 0.402 | 0.459 | - | 0.123 | 1.444 | 1.566 |
| *Amaranthus retroflexus* | 0.133 | 0.055 | 0.269 | 0.457 | 0.244 | 0.067 | 0.715 | 1.026 |
| *Phyllanthus urinaria* | 0.204 | 0.165 | 0.086 | 0.456 | 0.463 | 0.522 | 0.162 | 1.148 |
| *Ammannia baccifera* | 0.446 | - | - | 0.446 | 0.077 | - | - | 0.077 |
| *Erigeron annuus* | 0.038 | 0.236 | 0.171 | 0.445 | 0.077 | 0.071 | 0.138 | 0.286 |
| *Salvia plebeia* | 0.209 | 0.050 | 0.124 | 0.383 | 0.161 | 0.100 | 0.258 | 0.519 |
| *Portulaca oleracea* | 0.252 | 0.073 | - | 0.325 | 0.440 | 0.122 | - | 0.562 |
| *Pouzolzia zeylanica* | - | 0.023 | 0.275 | 0.298 | - | 0.067 | 0.564 | 0.631 |
| *Artemisia carvifolia* | - | 0.112 | 0.182 | 0.295 | - | 0.148 | 0.430 | 0.579 |
| *Anemarrhena asphodeloides* | - | 0.067 | 0.197 | 0.265 | - | 0.071 | 0.452 | 0.524 |
| *Physalis alkekengi* | 0.042 | 0.147 | 0.063 | 0.252 | 0.067 | 0.208 | 0.125 | 0.400 |
| *Vigna radiata* | 0.139 | - | 0.108 | 0.248 | 0.083 | - | 0.125 | 0.208 |
| *Arachis hypogaea* | - | - | 0.211 | 0.211 | - | - | 0.091 | 0.091 |
| *Aster tataricus* | 0.063 | - | 0.127 | 0.190 | 0.139 | - | 0.267 | 0.406 |
| *Lindernia procumbens* | 0.169 | - | - | 0.169 | 0.056 | - | - | 0.056 |
| *Chenopodium ambrosioides* | 0.116 | 0.045 | - | 0.161 | 0.215 | 0.071 | - | 0.286 |
| *Mazus japonicus* | - | - | 0.161 | 0.161 | - | - | 0.071 | 0.071 |
| *Ambrosia artemisiifolia* | - | 0.061 | 0.095 | 0.156 | - | 0.204 | 0.333 | 0.537 |
| *Alopecurus aequalis* | - | - | 0.152 | 0.152 | - | - | 0.090 | 0.090 |
| *Sesbania cannabina* | - | 0.123 | - | 0.123 | - | 0.421 | - | 0.421 |
| *Hemistepta lyrata* | - | - | 0.107 | 0.107 | - | - | 0.167 | 0.167 |
| *Vetiveria zizanioides* | - | - | 0.107 | 0.107 | - | - | 0.368 | 0.368 |
| *Lindernia crustacea* | 0.054 | - | 0.045 | 0.099 | 0.077 | - | 0.167 | 0.244 |
| *Bupleurum longiradiatum* | - | 0.091 | - | 0.091 | - | 0.067 | - | 0.067 |
| *Dactyloctenium aegyptium* | - | 0.087 | - | 0.087 | - | 0.271 | - | 0.271 |
| *Juncus effusus* | - | 0.083 | - | 0.083 | - | 0.091 | - | 0.091 |
| *Phyla nodiflora* | - | 0.082 | - | 0.082 | - | 0.091 | - | 0.091 |
| *Celosia argentea* | - | - | 0.080 | 0.080 | - | - | 0.196 | 0.196 |
| *Sorghum bicolor* | - | - | 0.072 | 0.072 | - | - | 0.139 | 0.139 |
| *Daucus carota* | - | - | 0.072 | 0.072 | - | - | 0.416 | 0.416 |
| *Leptochloa chinensis* | - | - | 0.071 | 0.071 | - | - | 0.318 | 0.318 |
| *Pilea cavaleriei* | - | - | 0.067 | 0.067 | - | - | 0.071 | 0.071 |
| *Cosmos bipinnata* | - | 0.063 | - | 0.063 | - | 0.056 | - | 0.056 |
| *Mosla scabra* | - | - | 0.058 | 0.058 | - | - | 0.167 | 0.167 |
| *Torulinium ferax* | 0.057 | - | - | 0.057 | 0.077 | - | - | 0.077 |
| *Artemisia capillaris* | - | - | 0.056 | 0.056 | - | - | 0.067 | 0.067 |
| *Sida acuta* | 0.050 | - | - | 0.050 | 0.083 | - | - | 0.083 |
| *Euphorbia hypericifolia* | - | - | 0.049 | 0.049 | - | - | 0.103 | 0.103 |
| *Arthraxon hispidus* | - | - | 0.042 | 0.042 | - | - | 0.125 | 0.125 |
| *Trigonotis peduncularis* | - | - | 0.036 | 0.036 | - | - | 0.071 | 0.071 |
| *Mimosa pudica* | - | - | 0.035 | 0.035 | - | - | 0.071 | 0.071 |
| *Rorippa indica* | 0.033 | - | - | 0.033 | 0.077 | - | - | 0.077 |
| *Leucaena leucocephala* | 0.029 | - | - | 0.029 | 0.077 | - | - | 0.077 |
| *Commelina communis* | - | - | 0.028 | 0.028 | - | - | 0.067 | 0.067 |
| *Vicia sepium* | - | - | 0.025 | 0.025 | - | - | 0.071 | 0.071 |
| *Medicago sativa* | - | - | 0.023 | 0.023 | - | - | 0.071 | 0.071 |
| *Corydalis pallida* | - | 0.017 | - | 0.017 | - | 0.077 | - | 0.077 |
| *Euphorbia helioscopia* | - | 0.014 | - | 0.014 | - | 0.067 | - | 0.067 |
| *Cucumis sativus* | - | - | 0.011 | 0.011 | - | - | 0.056 | 0.056 |
| *Erigeron acer* | - | - | 0.007 | 0.007 | - | - | 0.056 | 0.056 |

“-”. Species was disappeared; P_i_. Importance value; B_i_. Niche breadth.

**Table** **S6** Names of dominant species at the altitude of 145-155m. The numbers in the figure represent plant species , with their families and genera attached.

| Number | Plant specie | Family | Genus |
| --- | --- | --- | --- |
| 1 | *Cynodon dactylon* | *[Poaceae](https://www.cfh.ac.cn/1322829.sp)* | *[Cynodon](https://www.cfh.ac.cn/48650.sp)* |
| 2 | *Cyperus rotundus* | *[Cyperaceae](http://www.cfh.ac.cn/636.sp)* | *[Cyperus](http://www.cfh.ac.cn/45700.sp)* |
| 3 | *Xanthium sibiricum* | *[Asteraceae](http://www.cfh.ac.cn/1322825.sp)* | *[Xanthium](http://www.cfh.ac.cn/43784.sp)* |
| 4 | *Polygonum hydropiper* | *[Polygonaceae](http://www.cfh.ac.cn/721.sp)* | *[Persicaria](http://www.cfh.ac.cn/1315227.sp)* |
| 5 | *Echinochloa crusgalli* | *[Poaceae](http://www.cfh.ac.cn/1322829.sp)* | *[Echinochloa](http://www.cfh.ac.cn/48878.sp)* |
| 6 | *Bidens pilosa* | *[Asteraceae](http://www.cfh.ac.cn/1322825.sp)* | *[Bidens](http://www.cfh.ac.cn/43593.sp)* |
| 7 | *Alternanthera philoxeroides* | *[Amaranthaceae](http://www.cfh.ac.cn/724.sp)* | *[Alternanthera](http://www.cfh.ac.cn/12176.sp)* |
| 8 | *Abutilon theophrasti* | *[Malvaceae](http://www.cfh.ac.cn/815.sp)* | *[Abutilon](http://www.cfh.ac.cn/14676.sp)* |
| 9 | *Bidens tripartita* | *[Asteraceae](http://www.cfh.ac.cn/1322825.sp)* | *[Bidens](http://www.cfh.ac.cn/43593.sp)* |
| 10 | *Eclipta prostrata* | *[Asteraceae](http://www.cfh.ac.cn/1322825.sp)* | *[Eclipta](http://www.cfh.ac.cn/43811.sp)* |
| 11 | *Ammannia baccifera* | *[Lythraceae](http://www.cfh.ac.cn/844.sp)* | *[Ammannia](http://www.cfh.ac.cn/24861.sp)* |
| 12 | *Acalypha australis* | *[Euphorbiaceae](http://www.cfh.ac.cn/789.sp)* | *[Acalypha](http://www.cfh.ac.cn/27128.sp)* |
| 13 | *Digitaria sanguinalis* | *[Poaceae](http://www.cfh.ac.cn/1322829.sp)* | *[Digitaria](http://www.cfh.ac.cn/48953.sp)* |
| 14 | *Setaria viridis* | *[Poaceae](http://www.cfh.ac.cn/1322829.sp)* | *[Setaria](http://www.cfh.ac.cn/48982.sp)* |
| 15 | *Cyperus difformis* | *[Cyperaceae](http://www.cfh.ac.cn/636.sp)* | *[Cyperus](http://www.cfh.ac.cn/45700.sp)* |
| 16 | *Portulaca oleracea* | *[Portulacaceae](http://www.cfh.ac.cn/728.sp)* | *[Portulaca](http://www.cfh.ac.cn/12201.sp)* |
| 17 | *Eleusine indica* | *[Poaceae](http://www.cfh.ac.cn/1322829.sp)* | *[Eleusine](http://www.cfh.ac.cn/48629.sp)* |
| 18 | *Solanum nigrum* | *[Solanaceae](http://www.cfh.ac.cn/895.sp)* | *[Solanum](http://www.cfh.ac.cn/31809.sp)* |
| 19 | *Salvia plebeia* | *[Lamiaceae](http://www.cfh.ac.cn/1322848.sp)* | *[Salvia](http://www.cfh.ac.cn/33787.sp)* |
| 20 | *Phyllanthus urinaria* | *[Phyllanthaceae](http://www.cfh.ac.cn/1717.sp)* | *[Phyllanthus](http://www.cfh.ac.cn/26865.sp)* |
| 21 | *Lindernia procumbens* | *[Linderniaceae](http://www.cfh.ac.cn/53089.sp)* | *[Lindernia](http://www.cfh.ac.cn/34898.sp)* |
| 22 | *Artemisia argyi* | *[Asteraceae](http://www.cfh.ac.cn/1322825.sp)* | *[Artemisia](http://www.cfh.ac.cn/42711.sp)* |
| 23 | *Aeschynomene indica* | *[Fabaceae](http://www.cfh.ac.cn/1122.sp)* | *[Aeschynomene](http://www.cfh.ac.cn/23473.sp)* |
| 24 | *Eriochloa villosa* | *[Poaceae](http://www.cfh.ac.cn/1322829.sp)* | *[Eriochloa](http://www.cfh.ac.cn/48918.sp)* |
| 25 | *Vigna radiata* | *[Fabaceae](http://www.cfh.ac.cn/1122.sp)* | *[Vigna](http://www.cfh.ac.cn/23376.sp)* |
| 26 | *Amaranthus retroflexus* | *[Amaranthaceae](http://www.cfh.ac.cn/724.sp)* | *[Amaranthus](http://www.cfh.ac.cn/12084.sp)* |
| 27 | *Humulus scandens* | *[Cannabaceae](http://www.cfh.ac.cn/1135.sp)* | *[Humulus](http://www.cfh.ac.cn/9708.sp)* |
| 28 | *Chenopodium ambrosioides* | *[Amaranthaceae](http://www.cfh.ac.cn/724.sp)* | *[Chenopodium](http://www.cfh.ac.cn/11732.sp)* |
| 29 | *Aster tataricus* | *[Asteraceae](http://www.cfh.ac.cn/1322825.sp)* | *[Aster](http://www.cfh.ac.cn/42089.sp)* |
| 30 | *Torulinium ferax* | *[Cyperaceae](http://www.cfh.ac.cn/636.sp)* | *[Torulinium](http://www.cfh.ac.cn/45843.sp)* |
| 31 | *Lindernia crustacea* | *[Linderniaceae](http://www.cfh.ac.cn/53089.sp)* | *[Lindernia](http://www.cfh.ac.cn/34898.sp)* |
| 32 | *Sida acuta* | *[Malvaceae](http://www.cfh.ac.cn/815.sp)* | *[Sida](http://www.cfh.ac.cn/14656.sp)* |
| 33 | *Physalis alkekengi* | *[Solanaceae](http://www.cfh.ac.cn/895.sp)* | *[Alkekengi](http://www.cfh.ac.cn/7240957.sp)* |
| 34 | *Erigeron annuus* | *[Asteraceae](http://www.cfh.ac.cn/1322825.sp)* | *[Erigeron](http://www.cfh.ac.cn/42485.sp)* |
| 35 | *Euphorbia humifusa* | *[Euphorbiaceae](http://www.cfh.ac.cn/789.sp)* | *[Euphorbia](http://www.cfh.ac.cn/27296.sp)* |
| 36 | *Ageratum conyzoides* | *[Asteraceae](http://www.cfh.ac.cn/1322825.sp)* | *[Ageratum](http://www.cfh.ac.cn/44155.sp)* |
| 37 | *Rorippa indica* | *[Brassicaceae](http://www.cfh.ac.cn/1322827.sp)* | *[Rorippa](http://www.cfh.ac.cn/16697.sp)* |
| 38 | *Leucaena leucocephala* | *[Fabaceae](http://www.cfh.ac.cn/1122.sp)* | *[Leucaena](http://www.cfh.ac.cn/22363.sp)* |


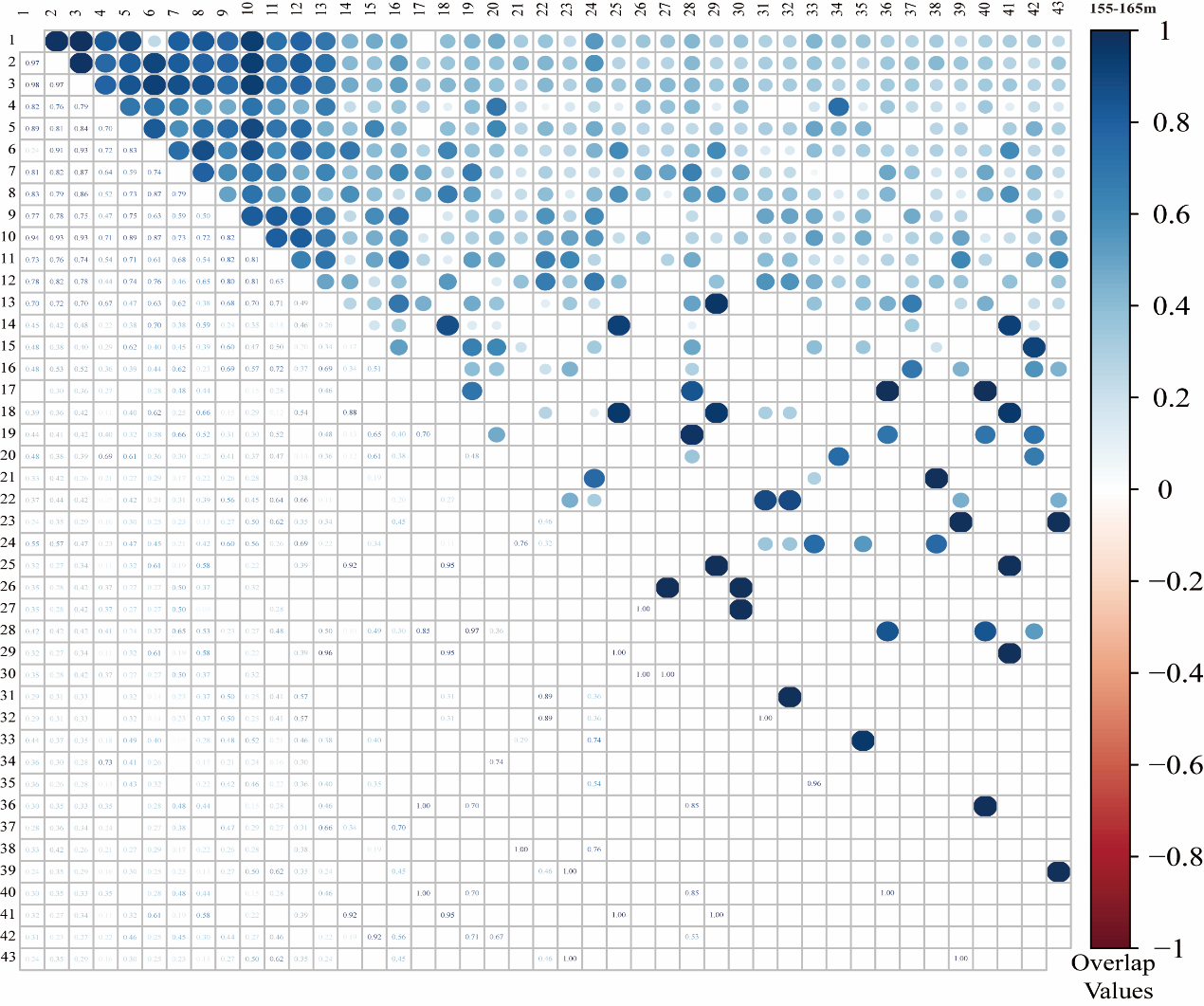


**Figure S1** Niche overlap values for dominant herbaceous plants in the 155-165 m altitude section in the HFZs. No.(1-43): plant species number (Please refer to supporting information Table S7 for complete plant species number).

**Table** **S7** Names of dominant species at the altitude of 155-165m. The numbers in the figure represent plant species , with their families and genera attached.

| Number | Plant specie | Family | Genus |
| --- | --- | --- | --- |
| 1 | *Cynodon dactylon* | *Poaceae* | *Cynodon* |
| 2 | *Xanthium sibiricum* | *Asteraceae* | *Xanthium* |
| 3 | *Echinochloa crusgalli* | *Poaceae* | *Echinochloa* |
| 4 | *Setaria viridis* | *Poaceae* | *Setaria* |
| 5 | *Cyperus rotundus* | *Cyperaceae* | *Cyperus* |
| 6 | *Bidens pilosa* | *Asteraceae* | *Bidens* |
| 7 | *Digitaria sanguinalis* | *Poaceae* | *Digitaria* |
| 8 | *Polygonum hydropiper* | *Polygonaceae* | *Persicaria* |
| 9 | *Abutilon theophrasti* | *Malvaceae* | *Abutilon* |
| 10 | *Eclipta prostrata* | *Asteraceae* | *Eclipta* |
| 11 | *Solanum nigrum* | *Solanaceae* | *Solanum* |
| 12 | *Acalypha australis* | *Euphorbiaceae* | *Acalypha* |
| 13 | *Bidens tripartita* | *Asteraceae* | *Bidens* |
| 14 | *Alternanthera philoxeroides* | *Amaranthaceae* | *Alternanthera* |
| 15 | *Eriochloa villosa* | *Poaceae* | *Eriochloa* |
| 16 | *Euphorbia humifusa* | *Euphorbiaceae* | *Euphorbia* |
| 17 | *Erigeron annuus* | *Asteraceae* | *Erigeron* |
| 18 | *Eleusine indica* | *Poaceae* | *Eleusine* |
| 19 | *Humulus scandens* | *Cannabaceae* | *Humulus* |
| 20 | *Melilotus officinalis* | *[Fabaceae](http://www.cfh.ac.cn/1122.sp)* | *[Melilotus](http://www.cfh.ac.cn/24473.sp)* |
| 21 | *Ageratum conyzoides* | *Asteraceae* | *Ageratum* |
| 22 | *Aeschynomene indica* | *Fabaceae* | *Aeschynomene* |
| 23 | *Cyperus difformis* | *Cyperaceae* | *Cyperus* |
| 24 | *Phyllanthus urinaria* | *Phyllanthaceae* | *Phyllanthus* |
| 25 | *Artemisia argyi* | *Asteraceae* | *Artemisia* |
| 26 | *Physalis alkekengi* | *Solanaceae* | *Alkekengi* |
| 27 | *Sesbania cannabina* | *[Fabaceae](http://www.cfh.ac.cn/1122.sp)* | *[Sesbania](http://www.cfh.ac.cn/22903.sp)* |
| 28 | *Artemisia carvifolia* | *[Asteraceae](http://www.cfh.ac.cn/1322825.sp)* | *[Artemisia](http://www.cfh.ac.cn/42711.sp)* |
| 29 | *Bupleurum longiradiatum* | *[Apiaceae](http://www.cfh.ac.cn/1322828.sp)* | *[Bupleurum](http://www.cfh.ac.cn/29841.sp)* |
| 30 | *Dactyloctenium aegyptium* | *[Poaceae](http://www.cfh.ac.cn/1322829.sp)* | *[Dactyloctenium](http://www.cfh.ac.cn/48632.sp)* |
| 31 | *Juncus effusus* | *[Juncaceae](http://www.cfh.ac.cn/661.sp)* | *[Juncus](http://www.cfh.ac.cn/45293.sp)* |
| 32 | *Phyla nodiflora* | *[Verbenaceae](http://www.cfh.ac.cn/893.sp)* | *[Phyla](http://www.cfh.ac.cn/32640.sp)* |
| 33 | *Portulaca oleracea* | *Portulacaceae* | *Portulaca* |
| 34 | *Anemarrhena asphodeloides* | *[Asparagaceae](http://www.cfh.ac.cn/1613.sp)* | *[Anemarrhena](http://www.cfh.ac.cn/49832.sp)* |
| 35 | *Cosmos bipinnata* | *[Asteraceae](http://www.cfh.ac.cn/1322825.sp)* | *[Cosmos](http://www.cfh.ac.cn/43618.sp)* |
| 36 | *Ambrosia artemisiifolia* | *[Asteraceae](http://www.cfh.ac.cn/1322825.sp)* | *[Ambrosia](http://www.cfh.ac.cn/43770.sp)* |
| 37 | *Conyza canadensis* | *[Asteraceae](http://www.cfh.ac.cn/1322825.sp)* | *[Erigeron](http://www.cfh.ac.cn/42485.sp)* |
| 38 | *Amaranthus retroflexus* | *Amaranthaceae* | *Amaranthus* |
| 39 | *Salvia plebeia* | *Lamiaceae* | *Salvia* |
| 40 | *Chenopodium ambrosioides* | *Amaranthaceae* | *Chenopodium* |
| 41 | *Pouzolzia zeylanica* | *[Urticaceae](http://www.cfh.ac.cn/703.sp)* | *[Pouzolzia](http://www.cfh.ac.cn/10565.sp)* |
| 42 | *Corydalis pallida* | *[Papaveraceae](http://www.cfh.ac.cn/749.sp)* | *[Corydalis](http://www.cfh.ac.cn/9016.sp)* |
| 43 | *Euphorbia helioscopia* | *[Euphorbiaceae](http://www.cfh.ac.cn/789.sp)* | *[Euphorbia](http://www.cfh.ac.cn/27296.sp)* |


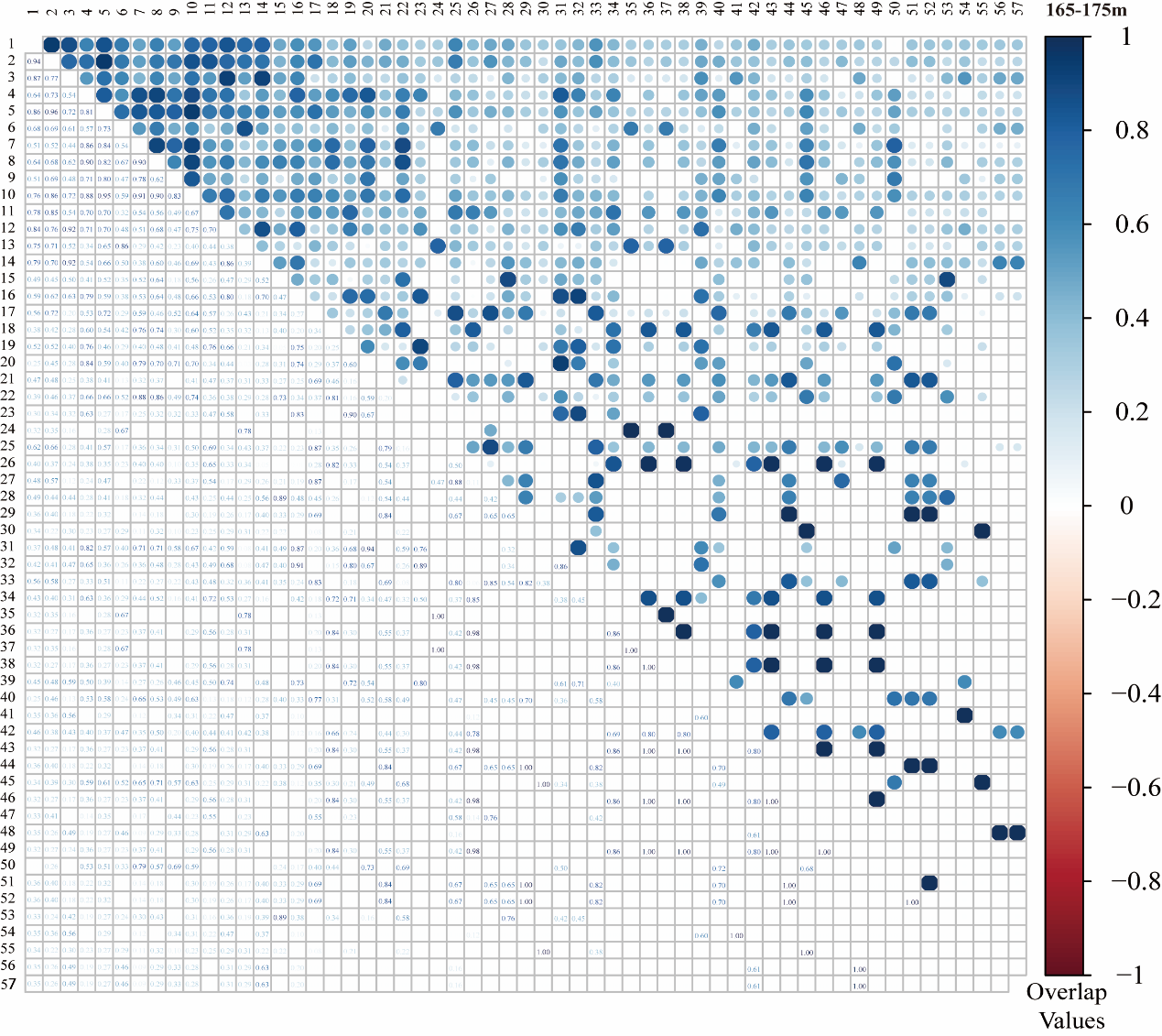


**Figure S2** Niche overlap values for dominant herbaceous plants in the 165-175 m altitude section in the HFZs. No.(1-57): plant species number (Please refer to supporting information Table S8 for complete plant species number).

**Table S8** Names of dominant species at the altitude of 165-175m. The numbers in the figure represent plant species , with their families and genera attached.

| Number | Plant specie | Family | Genus |
| --- | --- | --- | --- |
| 1 | *Cynodon dactylon* | *Poaceae* | *Cynodon* |
| 2 | *Xanthium sibiricum* | *Asteraceae* | *Xanthium* |
| 3 | *Setaria viridis* | *Poaceae* | *Setaria* |
| 4 | *Digitaria sanguinalis* | *Poaceae* | *Digitaria* |
| 5 | *Bidens pilosa* | *Asteraceae* | *Bidens* |
| 6 | *Melilotus officinalis* | *Fabaceae* | *Melilotus* |
| 7 | *Eclipta prostrata* | *Asteraceae* | *Eclipta* |
| 8 | *Echinochloa crusgalli* | *Poaceae* | *Echinochloa* |
| 9 | *Eriochloa villosa* | *Poaceae* | *Eriochloa* |
| 10 | *Polygonum hydropiper* | *Polygonaceae* | *Persicaria* |
| 11 | *Alternanthera philoxeroides* | *Amaranthaceae* | *Alternanthera* |
| 12 | *Bidens tripartita* | *Asteraceae* | *Bidens* |
| 13 | *Cyperus rotundus* | *Cyperaceae* | *Cyperus* |
| 14 | *Abutilon theophrasti* | *Malvaceae* | *Abutilon* |
| 15 | *Solanum nigrum* | *Solanaceae* | *Solanum* |
| 16 | *Acalypha australis* | *Euphorbiaceae* | *Acalypha* |
| 17 | *Conyza canadensis* | *Asteraceae* | *Erigeron* |
| 18 | *Eleusine indica* | *Poaceae* | *Eleusine* |
| 19 | *Pouzolzia zeylanica* | *Urticaceae* | *Pouzolzia* |
| 20 | *Amaranthus retroflexus* | *Amaranthaceae* | *Amaranthus* |
| 21 | *Ageratum conyzoides* | *Asteraceae* | *Ageratum* |
| 22 | *Cyperus difformis* | *Cyperaceae* | *Cyperus* |
| 23 | *Arachis hypogaea* | *[Fabaceae](http://www.cfh.ac.cn/1122.sp)* | *[Arachis](http://www.cfh.ac.cn/23490.sp)* |
| 24 | *Anemarrhena asphodeloides* | *Asparagaceae* | *Anemarrhena* |
| 25 | *Artemisia argyi* | *Asteraceae* | *Artemisia* |
| 26 | *Humulus scandens* | *Cannabaceae* | *Humulus* |
| 27 | *Artemisia carvifolia* | *Asteraceae* | *Artemisia* |
| 28 | *Erigeron annuus* | *Asteraceae* | *Erigeron* |
| 29 | *Mazus japonicus* | *[Mazaceae](http://www.cfh.ac.cn/7267112.sp)* | *[Mazus](http://www.cfh.ac.cn/34958.sp)* |
| 30 | *Alopecurus aequalis* | *[Poaceae](http://www.cfh.ac.cn/1322829.sp)* | *[Alopecurus](http://www.cfh.ac.cn/48436.sp)* |
| 31 | *Euphorbia humifusa* | *Euphorbiaceae* | *Euphorbia* |
| 32 | *Aeschynomene indica* | *Fabaceae* | *Aeschynomene* |
| 33 | *Aster tataricus* | *[Asteraceae](http://www.cfh.ac.cn/1322825.sp)* | *[Aster](http://www.cfh.ac.cn/42089.sp)* |
| 34 | *Salvia plebeia* | *Lamiaceae* | *Salvia* |
| 35 | *Vigna radiata* | *[Fabaceae](http://www.cfh.ac.cn/1122.sp)* | *[Vigna](http://www.cfh.ac.cn/23376.sp)* |
| 36 | *Hemistepta lyrata* | *[Asteraceae](http://www.cfh.ac.cn/1322825.sp)* | *[Hemisteptia](http://www.cfh.ac.cn/1312761.sp)* |
| 37 | *Vetiveria zizanioides* | *[Poaceae](http://www.cfh.ac.cn/1322829.sp)* | *[Chrysopogon](http://www.cfh.ac.cn/49211.sp)* |
| 38 | *Ambrosia artemisiifolia* | *Asteraceae* | *Ambrosia* |
| 39 | *Phyllanthus urinaria* | *Phyllanthaceae* | *Phyllanthus* |
| 40 | *Celosia argentea* | *[Amaranthaceae](http://www.cfh.ac.cn/724.sp)* | *[Celosia](http://www.cfh.ac.cn/12065.sp)* |
| 41 | *Sorghum bicolor* | *[Poaceae](http://www.cfh.ac.cn/1322829.sp)* | *[Sorghum](http://www.cfh.ac.cn/49190.sp)* |
| 42 | *Daucus carota* | *[Apiaceae](http://www.cfh.ac.cn/1322828.sp)* | *[Daucus](http://www.cfh.ac.cn/30461.sp)* |
| 43 | *Leptochloa chinensis* | *[Poaceae](http://www.cfh.ac.cn/1322829.sp)* | *[Leptochloa](http://www.cfh.ac.cn/48616.sp)* |
| 44 | *Pilea cavaleriei* | *[Urticaceae](http://www.cfh.ac.cn/703.sp)* | *[Pilea](http://www.cfh.ac.cn/10368.sp)* |
| 45 | *Physalis alkekengi* | *Solanaceae* | *Alkekengi* |
| 46 | *Mosla scabra* | *[Lamiaceae](http://www.cfh.ac.cn/1322848.sp)* | *[Mosla](http://www.cfh.ac.cn/34062.sp)* |
| 47 | *Artemisia capillaris* | *[Asteraceae](http://www.cfh.ac.cn/1322825.sp)* | *[Artemisia](http://www.cfh.ac.cn/42711.sp)* |
| 48 | *Euphorbia hypericifolia* | *[Euphorbiaceae](http://www.cfh.ac.cn/789.sp)* | *[Euphorbia](http://www.cfh.ac.cn/27296.sp)* |
| 49 | *Lindernia crustacea* | *[Linderniaceae](http://www.cfh.ac.cn/53089.sp)* | *[Lindernia](http://www.cfh.ac.cn/34898.sp)* |
| 50 | *Arthraxon hispidus* | *[Poaceae](http://www.cfh.ac.cn/1322829.sp)* | *[Arthraxon](http://www.cfh.ac.cn/49299.sp)* |
| 51 | *Trigonotis peduncularis* | *[Boraginaceae](http://www.cfh.ac.cn/892.sp)* | *[Trigonotis](http://www.cfh.ac.cn/32326.sp)* |
| 52 | *Mimosa pudica* | *[Fabaceae](http://www.cfh.ac.cn/1122.sp)* | *[Mimosa](http://www.cfh.ac.cn/22357.sp)* |
| 53 | *Commelina communis* | *[Commelinaceae](http://www.cfh.ac.cn/654.sp)* | *[Commelina](http://www.cfh.ac.cn/45201.sp)* |
| 54 | *Vicia sepium* | *[Fabaceae](http://www.cfh.ac.cn/1122.sp)* | *[Vicia](http://www.cfh.ac.cn/24371.sp)* |
| 55 | *Medicago sativa* | *[Fabaceae](http://www.cfh.ac.cn/1122.sp)* | *[Medicago](http://www.cfh.ac.cn/24492.sp)* |
| 56 | *Cucumis sativus* | *[Cucurbitaceae](http://www.cfh.ac.cn/915.sp)* | *[Cucumis](http://www.cfh.ac.cn/15383.sp)* |
| 57 | *Erigeron acer* | *[Asteraceae](http://www.cfh.ac.cn/1322825.sp)* | *[Erigeron](http://www.cfh.ac.cn/42485.sp)* |
